# Resilience among refugee mothers: scoping review of promotion and hindrance factors

**DOI:** 10.1192/bjo.2025.10781

**Published:** 2025-08-07

**Authors:** Francesca Zecchinato, Ingi Iusmen, Dawn-Marie Walker, Pia Riggirozzi, Ken Brackstone

**Affiliations:** Centre for Innovation in Mental Health, School of Psychology, Faculty of Environmental and Life Sciences, University of Southampton, Southampton, UK; Department of Politics and International Relations, School of Economic, Social and Political Sciences, Faculty of Social Sciences, University of Southampton, Southampton, UK; School of Health Sciences, Faculty of Environmental and Life Sciences, University of Southampton, Southampton, UK; Clinical Informatics Research Unit, Faculty of Medicine, University of Southampton, Southampton, UK

**Keywords:** Mothers, hindrance, promotion, refugees, resilience

## Abstract

**Background:**

Refugee mothers represent a significant proportion of the migrant population worldwide. Their resilience has important implications for their health and the positive adjustment of their family units. However, refugee mothers have received little attention in research.

**Aims:**

This review provides an overview of factors that may promote or hinder resilience among refugee mothers and a foundation for identifying potential targets for clinical and policy interventions.

**Method:**

A scoping review was conducted according to the Preferred Reporting Items for Systematic Reviews and Meta-Analyses Extension for Scoping Reviews (PRISMA-ScR) reporting guidelines, using pre-defined criteria and a relevant search strategy on four databases: Web of Science Core Collection, APA PsycINFO, Ovid Medline, and Ovid Embase Classic+Embase. Study characteristics and data on resilience promotion and hindrance factors were extracted, and results were narratively synthesised.

**Results:**

Five articles met our inclusion criteria. Four studies described resilience promotion factors, and two studies described resilience hindrance factors. External (social or instrumental, community or professional, economic, and cultural) and internal (individual or psychological, and spiritual or religious) resilience resources were perceived as important for building resilience among refugee mothers.

**Conclusions:**

The most recurrent resilience promotion factors related to possessing strong social networks and instrumental support, while the most recurrent resilience hindrance factors related to community and professional stressors, such as accessing healthcare. These findings serve as a first step towards identifying potential clinical and policy intervention targets to strengthen resilience in refugee mothers – a vulnerable and currently under-studied population. This review can provide a guide for policymakers, health professionals, refugee charities and local communities in prioritising the efforts to address refugee mothers’ needs.

Globally, there were around 281 million international migrants and refugees in the world by the end of 2023, which equated to 2.3% of the global population.^1^ Of these, 117.3 million were internally displaced and 43.4 million identified as refugees. The 1951 Refugee Convention defines a refugee as a person who, ‘owing to well-founded fear of being persecuted for reasons of race, religion, nationality, membership of a particular social group or political opinion, is outside the country of [their] nationality, and is unable or, owing to such fear, is unwilling to avail [themself] of the protection of that country’.^2^ Refugees are often forced to flee their home country due to persecution, conflict, violence, or human rights violations. Unlike migrants who move willingly from one country to another for reasons such as employment, education and better living conditions, refugees cannot safely return to their home countries.^2^

Forced displacement can pose significant life-threatening challenges and barriers to refugees,^3^ and over 63 000 refugees died or were reported missing globally in the last 10 years.^4^ Thus, refugees represent a vulnerable population that is systematically found to be at a much higher risk of adverse physical and mental health conditions.^5,6^ Indeed, refugees often experience difficulties in accessing health insurance, employment, adequate housing, and healthcare services. The stress of displacement to a new country is further compounded by a range of difficulties, including feelings of insecurity and isolation (e.g. due to language barriers), self-perceptions of affective deprivation, homesickness, strangeness in relation to new cultural habits, linguistic and religious differences, and hostility and discrimination received in host countries.^7^ Together, these difficulties make refugee populations particularly vulnerable to adverse mental health outcomes, such as depression and post-traumatic stress disorder (PTSD).^8–10^

In 2023, women and girls accounted for nearly half of global international refugees.^11^ The process of migration and resettlement can be especially challenging for refugee women travelling with children or those who are pregnant. This group represents a significant proportion of the refugee population and faces a range of unique challenges, including family separation, psychosocial stress, trauma, physical harm and injury, risks of exploitation and gender-based violence, and pregnancy-related health concerns.^11^ The stressors of raising children, coping with pregnancy, or giving birth in a new environment are likely to be amplified in the face of linguistic, cultural, social and financial struggles.^12^ Single mothers, especially, experience unique challenges as they attempt to balance the demands of paid work – fulfilling their new role as the breadwinner – and ongoing family obligations.^13^ These circumstances can negatively impact the mental health of mothers and, in turn, the well-being of the whole family unit.^14,15^ Thus, a stressful environment may have long-term implications and negatively affect the mental health of the children of refugee mothers, as well as their linguistic and psychosocial development.^16^

Research with high-risk populations, including refugees, highlights that not all trauma-exposed individuals experience mental health difficulties or family dysfunction. Resilience theory provides a framework for understanding this variability, emphasising resilience as a dynamic and multidimensional process rather than a fixed trait. The theory proposes that some individuals adapt and thrive despite adversity by leveraging protective factors across multiple ecological levels.^17^ Indeed, some individuals cope with and adapt to their new circumstances better than expected, particularly given their level of trauma exposure and difficulties previously experienced.^18^ Resilience, defined as the ability to adapt and thrive despite experiencing adversity, trauma or significant stress, is especially important for refugee mothers. The ability of mothers to ‘bounce back’ in the face of adversity can significantly affect the mental health and development of their children.^15^ Resilience theory emphasises that resilience emerges from the dynamic interplay of external resources, such as social support (family and friends, community networks), access to education, employment and healthcare, as well as individual resources, including psychological strengths (optimism and coping strategies), personal attributes (adaptability, a sense of meaning or purpose) and spiritual beliefs.^19–22^ These interacting factors may influence a refugee mother’s ability to recover from significant stress or trauma. For example, resilience can help refugees mitigate the adverse impacts of multilayer stressors on their health (e.g. discrimination or racism^23^) and alleviate adverse mental health outcomes such as depression.^24^

Previous reviews exploring resilience among displaced populations have focused primarily on children and adolescents^25–28^ or families,^29^ while mothers have received comparatively less attention in research. Nevertheless, mothers represent a unique refugee population. Their resilience can significantly impact their own mental and physical health, as well as contribute to the positive adjustment of their children and families,^15^ especially in situations where childcare-related stressors may be amplified by unfamiliar and constantly changing environments. Thus, shedding light on specific ways in which resilience can be promoted or hindered among refugee mothers can help identify specific intervention targets that may improve the migration experience and, subsequently, the mental health and well-being of refugee mothers and their families.

## Rationale

Despite the central role that mothers play in refugee family systems, there remains a critical gap in research examining the specific factors that influence their capacity to adapt and thrive in host countries. Most resilience research to date has focused on children, adolescents or general family units, overlooking the unique vulnerabilities and strengths of refugee mothers.^25–29^ This gap limits the development of targeted support structures and risks marginalising a key demographic in both research and policy efforts. This scoping review provides an overview of specific factors that may promote or hinder resilience among refugee mothers during transit, arrival and resettlement. A scoping review was selected as it was well-equipped to map the available evidence in the field and allowed the identification of key promotion and hindrance factors of resilience within the literature. The review focused on women aged 18 years and older with one or more children, or pregnant women, to better understand the subsequent contextual determinants of resilience among refugee mothers, and to provide a foundation for potential intervention targets. Overall, this scoping review served to provide suggestions for policy- and decision-makers, health professionals and the charity sector, and to contribute insights towards multiple academic disciplines, including public and mental health, migration, sociology and social work, and gender studies.

## Objectives

The main objectives of the present scoping review were to: (a) identify specific promotion and hindrance factors to resilience among refugee mothers during their transition to a host country (i.e. during transit, arrival and resettlement), including knowledge gaps for future research, and (b) provide a foundation for possible intervention targets, which could aid those in charge of policy implementation within community settings that host refugees during arrival and integration. Grounded in resilience theory, we strived to categorise resilience promotion and hindrance factors into external and internal domains. This theory served as the guiding framework for analysing and reporting our findings, and to ensure a comprehensive understanding of the systemic influences on the resilience of refugee mothers.

## Method

A scoping review was conducted in line with the reporting guidelines described in the Preferred Reporting Items for Systematic Reviews and Meta-Analyses Extension for Scoping Reviews (PRISMA-ScR).^30^ The protocol for this review was pre-registered and published on Open Science Framework before starting the screening process.^31^

### Search strategy

The search strategy was developed to be exhaustive in terms of identifying the existing literature investigating factors that promote and/or hinder resilience among refugee mothers. Four electronic bibliographic databases were searched in November 2023: Web of Science Core Collection, APA PsycINFO, Ovid Medline, and Ovid Embase Classic+Embase. Additionally, to identify as much relevant evidence as possible, we examined the reference lists of previous review papers which met our criteria. The database search was updated in April 2025.

We applied the following Boolean logic algorithm: (refugee* or ‘asylum seeker*’ or ‘forcibly displaced’ or migrant* or immigrant*) and (mother* or maternal* or parent* or female* or women) and (resilienc* or resilient or adaptation or hardiness or adjustment) and (promot* or protect* or enhanc* or foster* or risk* or barrier*). The search was limited to titles and abstracts. No time or language limits were applied. The full set of search strategies is available online in Supplement 1 available at https://doi.org/10.1192/bjo.2025.10781.

We used EndNote (version 20.2 for macOS; Clarivate, Philadelphia, PA, USA; https://www.endnote.com/) software and the Rayyan web app (for macOS; Rayyan Systems Inc., Doha, Qatar; https://rayyan.ai/ ) to manage, screen and review all suitable papers, and Accelerator (for macOS; Systematic Review Accelerator, Bond University, Queensland, Australia; https://sr-accelerator.com/) for the deduplication process.^32^

### Selection criteria

To be eligible for inclusion, we searched for primary research that: (a) focused on mothers classified as refugees aged 18 or older who had moved from one country to another, and (b) identified resilience promotion and/or hindrance factors. All qualitative and quantitative study designs were considered. Studies where outcomes were interpreted by the authors as resilience promotion or hindrance factors were included. Studies were required to be written in English.

All returned titles and abstracts were double-screened independently by two raters (F.Z. and K.B.: inter-rater agreement 90%) to check whether they met the pre-determined inclusion criteria. F.Z. and K.B. examined and resolved conflicts by consensus through discussion. F.Z. and K.B. then independently reviewed all retained full-texts (inter-rater agreement 99.5%), once again resolving disagreements by consensus through discussion.

### Data extraction

For the records that met the inclusion criteria at the full-text screening stage, data were extracted and charted independently by both F.Z. and K.B. to record the following information for each source: (a) authors and publication year, (b) total sample size, (c) country where the study was conducted, (d) characteristics of participants (age, nationality, race and/or ethnicity, marital status), (e) study design, (f) offspring age, (g) resilience promotion factors identified, (h) resilience hindrance factors identified and (i) effect sizes of associations between protective/hindering factors and resilience if reported (for quantitative studies only). Differences in extractions were handled by consensus.

### Data synthesis

Relevant promotion and hindrance factors were mapped and are presented within Tables 2 and 3. In line with the aims of a scoping review,^33,34^ a basic descriptive analysis and narrative overview is provided in relation to the research question.

## Results

**Fig. 1:**
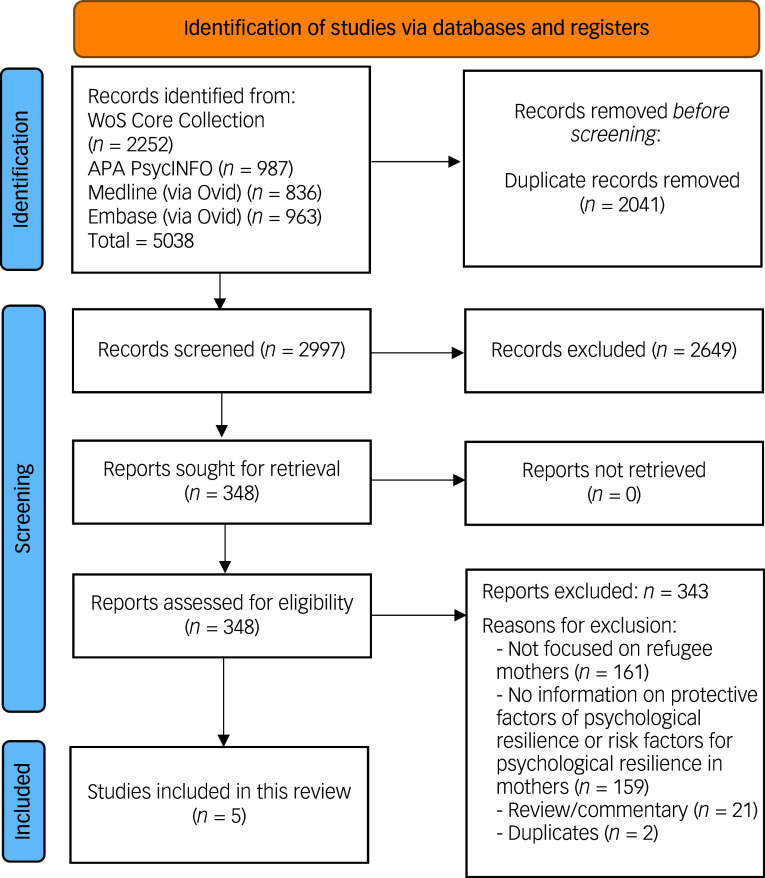
PRISMA flow diagram. Note. PRISMA, Preferred Reporting Items for Systematic Reviews and Meta-Analyses; WoS, Web of Science.

The database search resulted in 5038 citations, of which 2041 were duplicates. We screened 2997 abstracts, assessed 348 full-texts, and included 5 papers (see Fig. 1 for PRISMA flowchart). A list of the records excluded from this scoping review is included in Supplement 2, available online.

### Study characteristics

A total of five studies met the inclusion criteria and were included in the scoping review.^18,35–38^ Most studies explored refugee mothers only,^18,36–38^ and one study explored perspectives of both refugee mothers, fathers and refugee adolescents, although results were differentiated.^35^ Overall, included studies provided information on a total of 377 individual participants. Studies were published between 2015 and 2022. Most included studies were qualitative^35–38^ compared to quantitative.^18^ Sample sizes ranged between 8 and 28 for qualitative studies,^35–38^ and there was a sample size of 291 for the quantitative study.^18^ Most studies focused on refugees settling in Western countries.^18,35,37,38^ Two studies operationalised resilience^37,38^ or defined it during the paper,^37^ one study focused on adaptation or acculturation^37^ and one study did not operationalise or define resilience, but referred to it in interpreting key results.^35^ Table 1 provides a full overview of study characteristics.

**Table 1:** Characteristics of the included studies

Author (year)	Design	Country of the study	Ethnicity, race, origin	Mothers’ age	Mothers’ marital status	Sample size	Age of offspring
Betancourt et al (2015)^35^	Qualitative	USA	Somalia	N/A	N/A	*N* = 23	N/A
El-Khani et al (2017)^36^	Qualitative	Turkey	Syria	Range: 22–45 years	Not specified, although four were war widows	*N* = 27	N/A
Sim et al (2019)^18^	Quantitative	UK	Syria	Mean: 31.83 years (s.d. = 8.18; range: 19–56 years)	Married (91.4%), widowed (5.5%), in a relationship or separated/divorced (3.1%)	*N* = 291	Mean age: 7.44 years (s.d. = 3.23).
Tsai et al (2017)^37^	Qualitative	Australia	Burundi	Median: 39 years (range: 20–55)	Single (100%)	*N* = 8	Mean age of first child: 12 years (range: 2–28 years)
Zivot et al (2022)^38^	Qualitative	Canada	Angola, Congo, Eritrea, Ethiopia, India, Iraq, Nigeria, Sudan, Syria, Venezuela	N/A	Married (96%)	*N* = 28	N/A

N/A, not available.

### Resilience promotion and hindrance factors

All articles reported on resilience promotion (Table 2) and/or hindrance factors (Table 3) among refugee mothers. Within each factor, external and internal resilience resources were identified. This framework allowed for a comprehensive understanding of the multifaceted influences on resilience among refugee mothers.

**Table 2:** Factors promoting resilience among refugee mothers

Resource type	Theme (*n* of studies)	Author (year)	Promoting factors identified
External resources	Social resources (*n* = 4)	Betancourt et al (2015)^35^	Strong community networks and support Strong feelings of family unity, connectedness and communication
		El-Khani et al (2017)^36^	Social support and comfort-seeking from other mothers in a similar situation
		Sim et al (2019)^18^	Perceived feelings of emotional support from family members/friends
		Zivot et al (2022)^38^	Strong social support systems
	Community/professional resources (*n* = 1)	Zivot et al (2022)^38^	Participation in parental programmes focused on parent-involved learning for preschool age children
	Financial/economic resources (*n* = 1)	Zivot et al (2022)^38^	Access to spacious living environments Strong economic support systems
Internal resources	Individual resources (*n* = 2)	El-Khani et al (2017)^36^	Normalisation techniques (attempts to accept the situation, allowing themselves to normalise both their situation and changes seen in their children) Expressing gratitude and appreciation for things one still has, including health, children and family
		Zivot et al (2022)^38^	Holding optimistic mental attitudes and perspectives
	Faith (*n* = 2)	Betancourt et al (2015)^35^	Faith, religion and spirituality
		El-Khani et al (2017)^36^	

**Table 3:** Factors hindering resilience among refugee mothers

Resource type	Theme (*n* of studies)	Author (year)	Hindering factors identified
External resources	Community/professional stressors (*n* = 2)	Tsai et al (2017)^37^	Lack of appropriate knowledge of acceptable mothering practices, which led to the involvement of legal authorities who threatened to remove children from the mother’s care
		Zivot et al (2022)^38^	Experiencing challenges accessing healthcare
	Social stressors (*n* = 1)	Zivot et al (2022)^38^	Social isolation and concern for children’s mental health during pandemic
	Cultural stressors (*n* = 1)	Tsai et al (2017)^37^	Feeling that children acculturated to the new culture faster than themselves, which led to intergenerational gaps Inner conflicts associated with mothering practices, especially when mothers had arrived with a lack of knowledge relating to acceptable mothering practices in a new culture
	Financial/economic stressors (*n* = 1)	Zivot et al (2022)^38^	Experiencing disruptions to education, employment, income and spending

### Resilience promotion factors

From the four included studies that described factors promoting resilience among refugee mothers, five resilience promotion themes were identified. These were then grouped into external and internal resources.

#### External resources

Four studies identified social support and resources in the host country as a key resilience promotion factor.^18,35,36,38^ One study highlighted the importance of having access to community and/or professional services,^38^ and one study emphasised the importance of accessibility to economic resources and high-quality living environments.^38^

#### Internal resources

Two studies highlighted the importance of psychological strengths and individual factors,^36,38^ whereas two studies emphasised the importance of faith, religion and spirituality in promoting resilience.^35,36^

### Resilience hindrance factors

Four themes were identified in two studies that described resilience hindrance factors for refugee mothers, which were classified as external barriers.

#### External barriers

Two studies identified community stressors and lack of access to professional services as key hindrance factors among refugee mothers.^37,38^ These studies referred to challenges associated with healthcare access and a lack of knowledge regarding acceptable mothering practices in their new culture. One study emphasised the impact of family separation and social isolation during settlement in a new country,^38^ while another identified cultural stressors, including hardships associated with a new and unfamiliar culture.^37^ Finally, one study emphasised the relevance of financial and economic stressors among refugee mothers.^38^

## Discussion

This scoping review aimed to identify specific promotion and hindrance factors of resilience among refugee mothers during their transition and resettlement in a host country. Resilience theory, which emphasises the dynamic process through which individuals adapt and thrive despite adversity, provided a useful framework for understanding these factors.^17^ We found five articles that addressed our research question, identifying six themes related to factors important to resilience, which were further grouped into external or internal promotion and hindrance factors. These findings highlight potential targets for clinical and policy interventions and practice-related measures, as well as guidance to address the specific needs of refugee mothers. They are especially important given that refugee women are more likely to face unique challenges and protection risks during transit, arrival and resettlement.^11^ The stressors of raising children, coping with pregnancy or giving birth in a new environment are likely to be amplified in the face of linguistic, cultural, social and financial struggles.^12^ Indeed, the literature supports the development of culturally tailored, resilience-focused interventions that are sensitive to the unique stressors across the migration stages.^39^

### Promotion and hindrance factors

#### External resources

Refugee mothers highlighted access to social resources and connections as a key promotion factor of resilience. Maintaining social support with family members, friends and close others was deemed as paramount to coping with challenging circumstances during the migration period. This extended to support received from other refugee women and mothers, including those in the local population. Conversely, social isolation and an absence of social support was perceived as a key hindrance to resilience. These findings should encourage resources for more social and community programmes aimed at assisting refugee mothers to adapt to their new culture and social environment, especially mothers who may not have the familial support available due to physical separation. Such programmes would encourage support and communication about the specific challenges faced by refugee mothers, thus strengthening resilience during a stressful transition period.^12,40^

Access to community or professional support services was also identified as a key external resilience factor for refugee mothers. This referred to specific parenting services, such as professional advice in improving parent-involved learning for preschool age children. However, challenges pertaining to healthcare access and delays in receiving health coverage were perceived as a hindrance to resilience. Single specific complaints were directed toward child protection services and social workers, especially professionals who were perceived as unhelpful and unsympathetic. Improving access to community and professional services, such as healthcare and parenting services for refugee mothers, may assist in facilitating successful integration within the local community. Educational workshops and culturally sensitive training resources aimed at improving parent-focused learning may empower refugee mothers by equipping them with the necessary knowledge and skills to provide effective childcare in a new and unfamiliar environment. Successful integration therefore requires a social context that supports inclusion and participation.^41^

Financial and economic factors such as access to spacious accommodation facilities and strong economic support systems (e.g. financial support) were recognised as key for promoting the resilience of refugee mothers, while cultural stressors (e.g. intergenerational gaps and hardships related to a new and unfamiliar culture) were recognised as a hindrance to resilience. In this regard, access to multilingual information and assistance for navigating local services, including outreach programmes that educate refugee mothers about resources available to them, is crucial in promoting resilience and improving overall well-being. Accessible information and resources that clearly explain what cultural differences are likely to be encountered would also provide refugee mothers with a basic understanding of their new surroundings, thus supporting successful integration. Host countries should implement support programmes tailored for refugee mothers, for example childcare support for working mothers, which may help alleviate economic insecurity and strengthen among refugee mothers a sense of stability and resilience in the face of adversity.^42^

#### Internal resources

Internal resources included psychological resources such as expressing gratitude and appreciation for one’s health, children and family, as well as acceptance of, and familiarisation with, their new circumstances. Clinical programmes and policy interventions that strengthen the coping strategies of refugee mothers, including stress self-management and meaning-making through gratitude interventions, may be key to promoting resilience. In turn, targeted psychological support and culturally appropriate mental health services may help reduce risks associated with displacement-related stressors. These culturally adapted resilience-building programmes could be introduced in refugee community centres. Previous studies have demonstrated the effectiveness of such programmes, such as mindfulness techniques, among refugee populations.^43^ These services, if directed toward refugee mothers in Western cultures, may positively impact their ability to adapt and thrive in a new community and culture.

Further, encouragement of, and openness towards, the religion and religious beliefs of refugee mothers could facilitate successful integration within the local community.^44^ Accessible resources that help refugee mothers locate local places of worship relevant to their faith may be important in ensuring that they remain connected to their religious beliefs. Relatedly, strengthening healthcare access through community-based initiatives and faith-based networks may be particularly beneficial. Overall, these findings serve as evidence to guide the efforts of host countries to facilitate the resettlement process for refugee mothers, which can have important implications not only for their own mental and physical health, but also for the positive adjustment of their children and family units.^15^

Our results align closely with resilience theory, which posits that resilience is not a fixed trait, but rather a dynamic process shaped by external and internal resources.^19–22^ The contributing factors identified in this review reflect this conceptualisation and highlight that resilience may be bolstered through individual coping strategies and supportive environments. However, refugee mothers often face systemic barriers, including uncoordinated bureaucratic challenges, unemployment and financial hardships,^12^ which impact their resilience if these essential needs are unmet,^45^ hindering the effectiveness of interventions. Importantly, these barriers are often the result of restrictive policies in host countries that fail to support, if not actively undermine, the well-being of refugees – deterring asylum-seekers and undocumented migrants by limiting their access to housing, employment and public services. Such measures exacerbate the challenges faced by refugee mothers, making it even more difficult for them to build resilience. For example, a refugee mother who has developed strong coping mechanisms through engaging in social and community programmes may still struggle with resilience to stressors if she faces eviction or is unable to obtain legal work authorisation.^42,45,46^ Further, the responsibilities of raising children or navigating an unfamiliar healthcare system throughout pregnancy amplify these stressors,^47^ for which social support alone may be insufficient. Interventions should prioritise a holistic approach that combines resilience-building strategies with pragmatic policies aimed at addressing fundamental needs, such as stable housing and employment. Without securing these, efforts to enhance resilience will be futile, as ongoing structural insecurities would likely continue to increase stress and instability.^48^

However, while resilience theory offers a valuable framework for understanding how individuals adapt to adversity, it is also important to note that, when used uncritically, it may inadvertently place the burden of adaptation on individuals while obscuring the structural and systemic forces that produce vulnerability in the first place.^49^ For refugee mothers, framing challenges as requiring individual resilience may risk neglecting the role of unjust migration policies, social exclusion and systemic barriers such as poverty, housing insecurity and restricted access to healthcare.^50^ Additionally, some researchers argue that an overemphasis on resilience can lead to the expectation that individuals should remain strong or cope effectively regardless of context, potentially pathologising normal responses to chronic adversity or trauma.^51,52^ Thus, while our review identified key internal and external resources that foster resilience, we caution against viewing resilience as a substitute for structural change. A comprehensive approach should integrate resilience-building efforts with broader sociopolitical reforms that address the root causes of refugee vulnerability.

### Strengths, limitations and future directions

We conducted a scoping review that reflects the current state of the available literature on promoting and hindering factors of resilience among refugee mothers. We pre-registered our protocol and adhered to the PRISMA-ScR guidelines. The samples of the studies included provided some geographical diversity. Moreover, we set no limits for publication language or year, nor for study design. Crucially, we identified only five studies in the literature that discussed promotion and/or hindrance factors for resilience among refugee mothers. By highlighting the current paucity of studies focused on refugee mothers, this review underscores the need for future research to disaggregate refugee experiences by gender, caregiving role and family composition. By integrating findings with resilience theory and recognising the systemic challenges faced by refugee mothers, this review provides a framework for understanding resilience as more than an individual trait. Rather, it describes a complex interplay of interconnected external and internal factors that influences a refugee mother’s ability to recover from significant stress or trauma.

Overall, this review makes three key contributions to the literature. First, it synthesises and categorises resilience factors specifically for refugee mothers – a population rarely studied in isolation. Second, it applies resilience theory in a way that bridges individual-level and structural-level influences, offering a multilevel framework for intervention. Third, it identifies underexplored barriers such as institutional distrust and lack of culturally adapted mental health services, opening new avenues for policy and research. Importantly, this review also contributes to current debates on the intersectionality of forced migration, gender and mental health by focusing specifically on refugee mothers, an often-neglected population in resilience research (e.g.^28,29^). By disaggregating resilience into internal and external domains, this work provides a nuanced, ecologically grounded understanding of how resilience operates within systems shaped by both gendered vulnerability and institutional constraints.^53^

However, this review also has several limitations. First, only electronic academic databases were considered for our search. It is possible that other studies exploring resilience factors were published in the grey literature, such as governmental or technical reports published by countries hosting migrants and refugees. Such perspectives may have contributed to the themes and conclusions drawn from the study, and a wider mixture of host countries may have been included. Future research should consider perspectives outside of the peer-reviewed evidence base and attempt to integrate findings from across a broader range of disciplines, as well as publications from governmental and non-governmental sources. It is important to note, however, that a key strength of this study was its inclusion of non-peer reviewed research, such as PhD theses and student dissertations. Thus, some elements of grey literature were successfully integrated.

In addition, many of the included studies did not report the period in which the mothers arrived in the host country. This is crucial data to consider, since retrospective studies that tap into the resettlement experiences of mothers will likely contain inaccuracies, particularly if they suffered trauma during the migration period.^48^ Further, most studies did not provide detailed information regarding the sample being analysed, such as the reasons why the mothers left the country of origin, the specific circumstances in which mothers were living in, previous exposure to trauma and whether mothers travelled alone or with partners and/or family members. Crucially, these aspects are likely to impact the support needed to foster individual resilience and the adaptation process among refugee mothers, especially given the variety of reasons why they may have been forcibly displaced. This highlights the need for more research focused on the circumstances for displacement, the means of travel, the duration of the migration process and the diverse impact on resilience that this may entail among refugee mothers.

Further, the articles did not use a consistent definition or operationalisation of resilience. This inconsistency poses a key limitation, as it complicates the comparison of findings across studies and may hinder our understanding of resilience in refugee mothers. Adopting a standardised definition of resilience in future studies would enhance comparability and support the development of targeted interventions or policies. It is also important to note that we could not determine how many children were present at any stage during the migration period. Some mothers, for example, may have given birth after resettlement. Researchers exploring refugee parents should make greater efforts to collect child-related data, such as the number of children present during the migration process and the ages of these children. In this case, resilience may have been impacted by: (a) the number of children cared for during the transition, and (b) the ages of the children. For instance, different sources of support may be required for refugee mothers with preschool-age children compared to older children. Future research should attempt to explore these nuances in greater detail to allow the provision of more tailored support to this population group.

Finally, most studies focused on refugees settled in Western countries, with four of five included articles conducted in such settings. To generalise our findings, further research should examine resilience in other geographical areas. For example, Ghana has recently received an influx of refugees from Liberia and Burkina Faso.^54,55^ Reception conditions, asylum policies and available support may vary significantly across countries, and, thus, different contexts and circumstances (e.g. legal status, modes of travelling) may influence the specific facilitators and barriers to resilience among refugee mothers.

To conclude, this scoping review provides an overview of the existing literature on factors that promote or hinder psychological resilience among refugee mothers. Both external (social, community or professional, financial, and cultural) and internal (individual or psychological, and spiritual or religious) factors were identified as significant contributors to resilience. The most cited factor promoting resilience was the presence of a strong social and community network, including support from family, friends and relatives. In contrast, the most frequent hindrance to resilience involved challenges accessing healthcare and difficulties integrating into the local community. These findings are important because refugee women often face specific challenges and risks during transit, arrival and resettlement, with the stressors of raising children, coping with pregnancy or giving birth in a new environment likely to be amplified. Given that refugee women and their children will become future citizens in their host country and contribute to community and public life, it is essential that they receive support to build and maintain their resilience. Therefore, it is crucial that governments take action to mitigate the negative impact of resilience-hindering factors.

The findings from this review provide an initial step towards identifying key intervention targets (e.g. policy and clinical interventions, refugee support programmes) to promote resilience in refugee mothers – a vulnerable and currently understudied population. These insights offer an evidence-based resource to guide clinicians, policymakers, governments and local communities in addressing the needs of refugee mothers. Crucially, interventions targeting internal resilience may have limited effectiveness if fundamental needs such as stable housing, financial security and access to healthcare remain unmet. This underscores the necessity of adopting a holistic approach that integrates resilience-building efforts with policies designed to secure these essential needs. By addressing both individual and systemic barriers, governments and stakeholders can enhance the long-term well-being of refugee mothers and their families. This approach would foster not only their successful integration but also their contributions to society as future citizens.

## Supporting information

Zecchinato et al. supplementary material Zecchinato et al. supplementary material

## Data Availability

Data availability is not applicable to this article as no new data were created or analysed in this study.

## Supplementary material

The supplementary material is available online at https://doi.org/10.1192/bjo.2025.10781
