# Supplementary material for: Resilience among refugee mothers: scoping review of promotion and hindrance factors
Source: BJPsych Open. 2025 Aug 7;11(5):e172. doi: 10.1192/bjo.2025.10781 (PMC12451732; doi:10.1192/bjo.2025.10781)
Supplement: Zecchinato et al. supplementary material [file S2056472425107813sup001.docx]

Resilience among Refugee Mothers: A Scoping Review of Promotion and Hindrance Factors

Supplementary Material

**Table of Contents**

[Supplement 1. Full Search Strategy 4](#_Toc198915723)

[Supplement 2. Records Excluded from the Scoping Review 6](#_Toc198915724)

[Table S1: Records Excluded at the Full-text Screening Stage, with Reasons. 6](#_Toc198915725)

[Table S2: Records Excluded at the Full-text Screening Stage, with Reasons – Updated Search. 35](#_Toc198915726)

# Supplement 1. Full Search Strategy

1. **EBSCOhost APA PsycINFO**

(searched 04.11.2023)

S5

S1 AND S2 AND S3 AND S4

View Results (**883**)

S4

TI ( promot* or protect* or enhanc* or foster* or risk* or barrier* ) OR AB ( promot* or protect* or enhanc* or foster* or risk* or barrier* )

View Results (1,080,458)

S3

TI ( resilienc* or resilient or adaptation or hardiness or adjustment ) OR AB ( resilienc* or resilient or adaptation or hardiness or adjustment )

View Results (209,865)

S2

TI ( mother* or maternal* or parent* or female* or women ) OR AB ( mother* or maternal* or parent* or female* or women )

View Results (987,348)

S1

TI ( refugee* or "asylum seeker*" or "forcibly displaced" or migrant* or immigrant* ) OR AB ( refugee* or "asylum seeker*" or "forcibly displaced" or migrant* or immigrant* )

View Results (50,401)

**Search updated on 14.04.2025**

Limiters - Publication Date: 20231101-20250431 **🡪 104 additions**

1. **Ovid Medline**

(searched 04.11.2023)

<https://ovidsp.ovid.com/ovidweb.cgi?T=JS&NEWS=N&PAGE=main&SHAREDSEARCHID=28QIluvjBvA5Zfks6mOK9DLrGri2TXIemPjFhJ19pfiljUgR4pDDxRLawuBO7IsUk>

Ovid MEDLINE(R) ALL <1946 to November 03, 2023>

1 (refugee* or "asylum seeker*" or "forcibly displaced" or migrant* or immigrant*).ab,ti. 63198

2 (mother* or maternal* or parent* or female* or women).ab,ti. 2947581

3 (resilienc* or resilient or adaptation or hardiness or adjustment).ab,ti. 463832

4 (promot* or protect* or enhanc* or foster* or risk* or barrier*).ab,ti. 6719101

5 1 and 2 and 3 and 4 **718**

**Search updated on 14.04.2025**

limit 5 to dt=20231105-20250414 🡪 **118 additions**

1. **(OVID) Embase Classic + Embase**

(searched 04.11.2023)

<https://ovidsp.ovid.com/ovidweb.cgi?T=JS&NEWS=N&PAGE=main&SHAREDSEARCHID=6e6PEOZ8sqWmpgGaOsfOlItF4nUQ0Djcz2vgsI54R59b9hn1hJBpEY6NmLzRdLvFG>

Embase Classic+Embase <1947 to 2023 November 03>

1 (refugee* or "asylum seeker*" or "forcibly displaced" or migrant* or immigrant*).ab,ti. 71583

2 (mother* or maternal* or parent* or female* or women).ab,ti. 4348291

3 (resilienc* or resilient or adaptation or hardiness or adjustment).ab,ti. 599292

4 (promot* or protect* or enhanc* or foster* or risk* or barrier*).ab,ti. 8873671

5 1 and 2 and 3 and 4 832

**Search updated on 14.04.2025**

limit 5 to dc=20231105-20250414 🡪 **131 additions**

1. **Web of Science Core Collection**

(Searched 04.11.2023)

<https://www.webofscience.com/wos/woscc/summary/4e3ecc82-9582-4ab5-8b5a-d143bf9fd32d-b14291d5/relevance/1>

(((TS=(refugee* or "asylum seeker*" or "forcibly displaced" or migrant* or immigrant*)) AND TS=(mother* or maternal* or parent* or female* or women)) AND TS=(resilienc* or resilient or adaptation or hardiness or adjustment)) AND TS=(promot* or protect* or enhanc* or foster* or risk* or barrier*)

Search on Topic (includes Title, Abstract, keywords and keywords plus)

TOT. **1,916**

**Search updated on 14.04.2025**

search limited by index date (20231105-20250414) 🡪 **336 additions**

# Supplement 2. Records Excluded from the Scoping Review

## Table S1: Records Excluded at the Full-text Screening Stage, with Reasons.

| **Authors (Year)** | **Title** | **Exclusion reason** |
| --- | --- | --- |
| Abbey et al. (2023) | Perceived family support and student outcomes in rural china: A mediation analysis | No information on protective factors of psychological resilience or risk factors for psychological resilience in mothers |
| Abrams et al. (2022) | Fractured Families and Social Networks: Identifying Risk and Resilience Factors for Supporting Positive Mental Health in Venezuelan Immigrant Groups | Not focused on forcibly displaced mothers or information on mothers cannot be distinguished from the study sample (e.g., parents, offspring, women) |
| Abuzahra (2004) | Understanding resilience in Muslim-American immigrant women: An examination of protective processes | Not focused on forcibly displaced mothers or information on mothers cannot be distinguished from the study sample (e.g., parents, offspring, women) |
| Acharya and Northcott (2007) | Mental distress and the coping strategies of elderly Indian immigrant women | Not focused on forcibly displaced mothers or information on mothers cannot be distinguished from the study sample (e.g., parents, offspring, women) |
| Afifi et al. (2013) | The Relative Impacts of Uncertainty and Mothers' Communication on Hopelessness Among Palestinian Refugee Youth | No information on protective factors of psychological resilience or risk factors for psychological resilience in mothers |
| Afrin et al. (2019) | I Wish for My Daughters to Remain Under My 'Wings': An Exploration of Bangladeshi Immigrant Mothers' Socialisation Process of Raising Adolescent Daughters in Melbourne, Australia | No information on protective factors of psychological resilience or risk factors for psychological resilience in mothers |
| Aggarwal et al. (2014) | Using consumer perspectives to inform the cultural adaptation of psychological treatments for depression: A mixed methods study from South Asia | Not focused on forcibly displaced mothers or information on mothers cannot be distinguished from the study sample (e.g., parents, offspring, women) |
| Ahmad et al. (2013) | Resilience and Resources Among South Asian Immigrant Women as Survivors of Partner Violence | Not focused on forcibly displaced mothers or information on mothers cannot be distinguished from the study sample (e.g., parents, offspring, women) |
| Ahmad-Stout et al. (2018) | Experiences of Intimate Partner Violence: Findings From Interviews With South Asian Women in the United States | Not focused on forcibly displaced mothers or information on mothers cannot be distinguished from the study sample (e.g., parents, offspring, women) |
| Ahmed et al. (2008) | Experiences of immigrant new mothers with symptoms of depression | No information on protective factors of psychological resilience or risk factors for psychological resilience in mothers |
| Ahrne et al. (2022) | Group antenatal care (gANC) for Somali-speaking women in Sweden - a process evaluation | No information on protective factors of psychological resilience or risk factors for psychological resilience in mothers |
| Akesson and Sousa (2020) | Parental Suffering and Resilience Among Recently Displaced Syrian Refugees in Lebanon | Not focused on forcibly displaced mothers or information on mothers cannot be distinguished from the study sample (e.g., parents, offspring, women) |
| Akhtar et al. (2021) | Feasibility trial of a scalable transdiagnostic group psychological intervention for Syrians residing in a refugee camp | Not focused on forcibly displaced mothers or information on mothers cannot be distinguished from the study sample (e.g., parents, offspring, women) |
| Akinsulure-Smith et al. (2013) | Nah We Yone's De Fambul Camp: Facilitating Resilience in Displaced African Children | Not focused on forcibly displaced mothers or information on mothers cannot be distinguished from the study sample (e.g., parents, offspring, women) |
| Akintunde et al. (2023) | Adverse Childhood Experiences and Subjective Well-Being of Migrants: Exploring the Role of Resilience and Gender Differences | Not focused on forcibly displaced mothers or information on mothers cannot be distinguished from the study sample (e.g., parents, offspring, women) |
| Al-Krenawi and Bell (2023) | Gender differences in Syrian refugees in Jordan: Psychological, self-esteem, family function, marital satisfaction, and life satisfaction | No information on protective factors of psychological resilience or risk factors for psychological resilience in mothers |
| Alaazi et al. (2022) | Mobilizing communities and families for child mental health promotion in Canada: Views of African immigrants | Not focused on forcibly displaced mothers or information on mothers cannot be distinguished from the study sample (e.g., parents, offspring, women) |
| Alamdari et al. (2022) | Resilience factors among adults affected by mass conflict: Recommendations for researchers | Not focused on forcibly displaced mothers or information on mothers cannot be distinguished from the study sample (e.g., parents, offspring, women) |
| Alatorre (2012) | From criminalization to symbolic resiliency: Undocumented immigrants 're-imagining success' in the United States | Not focused on forcibly displaced mothers or information on mothers cannot be distinguished from the study sample (e.g., parents, offspring, women) |
| Alexander et al. (2021) | Post-migration Stressors and Subjective Well-Being in Adult Syrian Refugees Resettled in Sweden: A Gender Perspective | Not focused on forcibly displaced mothers or information on mothers cannot be distinguished from the study sample (e.g., parents, offspring, women) |
| Ali et al. (2012) | Assessment of prevalence and determinants of posttraumatic stress disorder in survivors of earthquake in Pakistan using Davidson Trauma Scale | Not focused on forcibly displaced mothers or information on mothers cannot be distinguished from the study sample (e.g., parents, offspring, women) |
| Alsharaydeh et al. (2023) | Challenges, coping and resilience in caring for children with disability among immigrant parents: A mixed methods study | No information on protective factors of psychological resilience or risk factors for psychological resilience in mothers |
| Alwan et al. (2019) | A qualitative study of the parental perceptions, beliefs, and behaviors that impact healthcare utilization of Syrian Refugee children | Not focused on forcibly displaced mothers or information on mothers cannot be distinguished from the study sample (e.g., parents, offspring, women) |
| Alwan et al. (2020) | Beliefs, perceptions, and behaviors impacting healthcare utilization of Syrian refugee children | Not focused on forcibly displaced mothers or information on mothers cannot be distinguished from the study sample (e.g., parents, offspring, women) |
| Anguiano and Lopez (2012) | El Miedo y El Hambre: Understanding the Familial, Social, and Educational Realities of Undocumented Latino Families in North Central Indiana | Not focused on forcibly displaced mothers or information on mothers cannot be distinguished from the study sample (e.g., parents, offspring, women) |
| Annan et al. (2016) | Improving Mental Health Outcomes of Burmese Migrant and Displaced Children in Thailand: a Community-Based Randomized Controlled Trial of a Parenting and Family Skills Intervention | Not focused on forcibly displaced mothers or information on mothers cannot be distinguished from the study sample (e.g., parents, offspring, women) |
| Antai (2010) | Social context, social position and child survival: Social determinants of child health inequities in Nigeria | No information on protective factors of psychological resilience or risk factors for psychological resilience in mothers |
| Antai et al. (2010) | Migration and child health inequities in Nigeria: a multilevel analysis of contextual- and individual-level factors | No information on protective factors of psychological resilience or risk factors for psychological resilience in mothers |
| Araya et al. (2011) | Quality of life after postconflict displacement in Ethiopia: comparing placement in a community setting with that in shelters | Not focused on forcibly displaced mothers or information on mothers cannot be distinguished from the study sample (e.g., parents, offspring, women) |
| Arce et al. (2020) | Tenemos que ser la voz': Exploring resilience among Latina/o immigrant families in the context of restrictive immigration policies and practices | Not focused on forcibly displaced mothers or information on mothers cannot be distinguished from the study sample (e.g., parents, offspring, women) |
| Arnetz et al. (2013) | Resilience as a Protective Factor Against the Development of Psychopathology Among Refugees | Not focused on forcibly displaced mothers or information on mothers cannot be distinguished from the study sample (e.g., parents, offspring, women) |
| Aroian and Norris (2000) | Resilience, stress, and depression among Russian immigrants to Israel | Not focused on forcibly displaced mothers or information on mothers cannot be distinguished from the study sample (e.g., parents, offspring, women) |
| Aroian et al. (2009) | A model of mother-child adjustment in Arab Muslim immigrants to the US | No information on protective factors of psychological resilience or risk factors for psychological resilience in mothers |
| Arrey et al. (2014) | "My virus, my healthcare": Perspectives of sub-saharan African migrant women with HIV/AIDS on treatment and care in Belgium | Not focused on forcibly displaced mothers or information on mothers cannot be distinguished from the study sample (e.g., parents, offspring, women) |
| Aryal et al. (2020) | The Impact of Spousal Migration on the Mental Health of Nepali Women: A Cross-Sectional Study | Not focused on forcibly displaced mothers or information on mothers cannot be distinguished from the study sample (e.g., parents, offspring, women) |
| Ascenso (2021) | Flourishing Through Music Creation: A Qualitative Investigation of the Lullaby Project Among Refugee and Incarcerated Communities | Not focused on forcibly displaced mothers or information on mothers cannot be distinguished from the study sample (e.g., parents, offspring, women) |
| Ataca (2002) | Psychological, sociocultural, and marital adaptation of Turkish immigrants in Canada | Not focused on forcibly displaced mothers or information on mothers cannot be distinguished from the study sample (e.g., parents, offspring, women) |
| Aube et al. (2019) | La Maison Bleue: Strengthening resilience among migrant mothers living in Montreal, Canada | Not focused on forcibly displaced mothers or information on mothers cannot be distinguished from the study sample (e.g., parents, offspring, women) |
| Ayón and Villa (2013) | Promoting Mexican Immigrant Families' Well-Being: Learning From Parents What Is Needed to Have a Strong Family | Not focused on forcibly displaced mothers or information on mothers cannot be distinguished from the study sample (e.g., parents, offspring, women) |
| Bahar (2015) | How do low-income Kurdish migrant women reconstruct their lives in an inner-city neighborhood of Istanbul?: Experiences of migration and adaptation to life in the city | No information on protective factors of psychological resilience or risk factors for psychological resilience in mothers |
| Baiden and Evans (2022) | Recruitment Strategies to Engage Newcomer Mothers of African Descent in Maternal Mental Health Research in Canada | No information on protective factors of psychological resilience or risk factors for psychological resilience in mothers |
| Baik et al. (2021) | Mothering and mothered during defection and resettlement: Experiences of North Korean refugee women and their children | No information on protective factors of psychological resilience or risk factors for psychological resilience in mothers |
| Baker (2009) | Promoting resilience: A program for children at risk for child maltreatment and their families | No information on protective factors of psychological resilience or risk factors for psychological resilience in mothers |
| Baker et al. (2019) | Optimising refugee children's health/wellbeing in preparation for primary and secondary school: a qualitative inquiry | Not focused on forcibly displaced mothers or information on mothers cannot be distinguished from the study sample (e.g., parents, offspring, women) |
| Barnes and Theule (2023) | Examining Associations between Maternal Trauma, Child Attachment Security, and Child Behaviours in Refugee Families | No information on protective factors of psychological resilience or risk factors for psychological resilience in mothers |
| Bayhan et al. (2022) | The study of the group intervention containing EMDR therapy for children and mothers in the field of trauma after a mine explosion in Turkey | No information on protective factors of psychological resilience or risk factors for psychological resilience in mothers |
| Bedos et al. (2004) | Use of prophylactic dental services by immigrant mothers in Quebec | Not focused on forcibly displaced mothers or information on mothers cannot be distinguished from the study sample (e.g., parents, offspring, women) |
| Beiser et al. (2011) | Stresses of Passage, Balms of Resettlement, and Posttraumatic Stress Disorder Among Sri Lankan Tamils in Canada | Not focused on forcibly displaced mothers or information on mothers cannot be distinguished from the study sample (e.g., parents, offspring, women) |
| Bender and Castro (2000) | Explaining the birth weight paradox: Latina immigrants' perceptions of resilience and risk | Not focused on forcibly displaced mothers or information on mothers cannot be distinguished from the study sample (e.g., parents, offspring, women) |
| Berckmoes and Mazzucato (2018) | Resilience among Nigerian transnational parents in the Netherlands: a strength-based approach to migration and transnational parenting | Not focused on forcibly displaced mothers or information on mothers cannot be distinguished from the study sample (e.g., parents, offspring, women) |
| Betancourt et al. (2012) | Caregiver and Adolescent Mental Health in Ethiopian Kunama Refugees Participating in an Emergency Education Program | No information on protective factors of psychological resilience or risk factors for psychological resilience in mothers |
| Bhuyan and Leung (2022) | Framing Migrant Resilience as a Civic Responsibility: A Case Study of Municipal and Provincial Immigrant Integration Policies in Toronto, Ontario | Not focused on forcibly displaced mothers or information on mothers cannot be distinguished from the study sample (e.g., parents, offspring, women) |
| Blanco and Otero García (2022) | Perceived facilitating and hindering factors to exclusive breastfeeding among Latin American immigrant women living in Colmenar Viejo (Community of Madrid, Spain) | No information on protective factors of psychological resilience or risk factors for psychological resilience in mothers |
| Blanco et al. (2015) | Characteristics of Caregiving Practices in Colombian Families with Children in Early Childhood in Forcibly Displaced Situation | No information on protective factors of psychological resilience or risk factors for psychological resilience in mothers |
| Bleich et al. (2006) | Mental health and resiliency following 44 months of terrorism: a survey of an Israeli national representative sample | Not focused on forcibly displaced mothers or information on mothers cannot be distinguished from the study sample (e.g., parents, offspring, women) |
| Brand et al. (2014) | Young African Female Refugees' Sense of Acculturation and Community Connection in Western Australia | Not focused on forcibly displaced mothers or information on mothers cannot be distinguished from the study sample (e.g., parents, offspring, women) |
| Bridi et al. (2023) | The Influences of Faith on Illness Representations and Coping Procedures of Mental and Cognitive Health Among Aging Arab Refugees: A Qualitative Study | Not focused on forcibly displaced mothers or information on mothers cannot be distinguished from the study sample (e.g., parents, offspring, women) |
| Bromand et al. (2010) | Mental health resilience and vulnerability factors in Turkish female migrants and German controls | Review, commentary papers and research protocols (i.e., not reporting novel data/findings) |
| Bromand et al. (2012) | Mental health of Turkish women in Germany: Resilience and risk factors | Not focused on forcibly displaced mothers or information on mothers cannot be distinguished from the study sample (e.g., parents, offspring, women) |
| Browne et al. (2021) | Refugee Children and Families During the COVID-19 Crisis: A Resilience Framework for Mental Health | Review, commentary papers and research protocols (i.e., not reporting novel data/findings) |
| Buhary et al. (2023) | Using intersectionality to explore resilience of pregnant migrant Sri Lankan women during the covid-19 lockdowns in Victoria, Australia: an intersectional qualitative study | No information on protective factors of psychological resilience or risk factors for psychological resilience in mothers |
| Bustos and Santiago (2023) | Effects of familism, parenting, and family cohesion on child internalizing symptoms among Mexican immigrant families | No information on protective factors of psychological resilience or risk factors for psychological resilience in mothers |
| Byers et al. (2022) | Improving Child Health and Healthcare Use Outcomes: How Risk and Resilience Intersect in Pediatric Care | Not focused on forcibly displaced mothers or information on mothers cannot be distinguished from the study sample (e.g., parents, offspring, women) |
| Byrskog et al. (2014) | Violence and reproductive health preceding flight from war: accounts from Somali born women in Sweden | Not focused on forcibly displaced mothers or information on mothers cannot be distinguished from the study sample (e.g., parents, offspring, women) |
| Byrskog et al. (2016) | Moving on' Violence, wellbeing and questions about violence in antenatal care encounters. A qualitative study with Somali-born refugees in Sweden | Not focused on forcibly displaced mothers or information on mothers cannot be distinguished from the study sample (e.g., parents, offspring, women) |
| Calzada et al. (2009) | Parent cultural adaptation and child functioning in culturally diverse, urban families of preschoolers | Not focused on forcibly displaced mothers or information on mothers cannot be distinguished from the study sample (e.g., parents, offspring, women) |
| Campbell (2008) | Lessons in resilience - Undocumented Mexican women in South Carolina | Not focused on forcibly displaced mothers or information on mothers cannot be distinguished from the study sample (e.g., parents, offspring, women) |
| Cardoso et al. (2022) | Migrant mothers' and youths' experiences of separation and reunification | Review, commentary papers and research protocols (i.e., not reporting novel data/findings) |
| Carmody et al. (2021) | Narratives of Women-at-Risk Resettled in Australia: Loss, Renewal and Connection | Not focused on forcibly displaced mothers or information on mothers cannot be distinguished from the study sample (e.g., parents, offspring, women) |
| Carpenter (2013) | Family in the Borderlands/LA Frontera: Transnational narratives of Mexican migrant parents and their young children | Not focused on forcibly displaced mothers or information on mothers cannot be distinguished from the study sample (e.g., parents, offspring, women) |
| Carter et al. (2022) | Adult newcomers' perceptions of access to care and differences in health systems after relocation from Syria | Not focused on forcibly displaced mothers or information on mothers cannot be distinguished from the study sample (e.g., parents, offspring, women) |
| Casas et al. (2010) | "Se vale llorar y se vale reir": Latina Immigrants' Coping Strategies for Maintaining Mental Health in the Face of Immigration-Related Stressors | Not focused on forcibly displaced mothers or information on mothers cannot be distinguished from the study sample (e.g., parents, offspring, women) |
| Castro (2022) | Tears, trauma and transformation: Central American mothers' experiences of violence, migration and family reunification | No information on protective factors of psychological resilience or risk factors for psychological resilience in mothers |
| Cataudella et al. (2012) | Migrazione: Minori e genitori tra vulnerabilità e potenzialità = Migration: Children, adolescents and parents between vulnerability and resilience | Review, commentary papers and research protocols (i.e., not reporting novel data/findings) |
| Ceballo et al. (2020) | Contextual stressors and the role of religion and spirituality in the mental health of Latino/a immigrant parents and youth | Review, commentary papers and research protocols (i.e., not reporting novel data/findings) |
| Cenek et al. (2015) | Czech citizens in Zambia: Preliminary research on the process of adaptation | Not focused on forcibly displaced mothers or information on mothers cannot be distinguished from the study sample (e.g., parents, offspring, women) |
| Cerdeña (2021) | Onward: An ethnography of Latina migrant motherhood during the COVID-19 pandemic | No information on protective factors of psychological resilience or risk factors for psychological resilience in mothers |
| Cesario et al. (2014) | Functioning outcomes for abused immigrant women and their children 4 months after initiating intervention | No information on protective factors of psychological resilience or risk factors for psychological resilience in mothers |
| Çetrez et al. (2021) | A Public Mental Health Study Among Iraqi Refugees in Sweden: Social Determinants, Resilience, Gender, and Cultural Context | Not focused on forcibly displaced mothers or information on mothers cannot be distinguished from the study sample (e.g., parents, offspring, women) |
| Chang (2000) | Listening to well-educated Chinese immigrant families: The parents' perceptions of family resilience | Not focused on forcibly displaced mothers or information on mothers cannot be distinguished from the study sample (e.g., parents, offspring, women) |
| Chang et al. (2000) | The perception of resiliency mechanisms in Chinese American families: Implications for family therapy | Not focused on forcibly displaced mothers or information on mothers cannot be distinguished from the study sample (e.g., parents, offspring, women) |
| Chavez (2017) | It's all in the Raiz: Mexican immigrant mothers' perception of academic success | No information on protective factors of psychological resilience or risk factors for psychological resilience in mothers |
| Chen et al. (2022) | Bi-dimensional acculturation and social support on perinatal depression in marriage-based immigrant women | No information on protective factors of psychological resilience or risk factors for psychological resilience in mothers |
| Chen et al. (2022) | Psychological distress among immigrant women who divorced: Resilience as a mediator | Not focused on forcibly displaced mothers or information on mothers cannot be distinguished from the study sample (e.g., parents, offspring, women) |
| Chernet et al. (2021) | Mental health and resilience among Eritrean refugees at arrival and one-year post-registration in Switzerland: a cohort study | Not focused on forcibly displaced mothers or information on mothers cannot be distinguished from the study sample (e.g., parents, offspring, women) |
| Cho et al. (2017) | Culturally Adaptive Walking Intervention for Korean-Chinese Female Migrant Workers | Not focused on forcibly displaced mothers or information on mothers cannot be distinguished from the study sample (e.g., parents, offspring, women) |
| Cho et al. (2018) | Acculturation, Acculturative Stress, and Depressive Symptoms in International Migrants: A Study with Vietnamese Women in South Korea | Not focused on forcibly displaced mothers or information on mothers cannot be distinguished from the study sample (e.g., parents, offspring, women) |
| Choi et al. (2009) | Acculturation and Depressive Symptoms in Korean Immigrant Women | Not focused on forcibly displaced mothers or information on mothers cannot be distinguished from the study sample (e.g., parents, offspring, women) |
| Choi et al. (2014) | The experience of Korean immigrant women adjusting to Canadian society | Not focused on forcibly displaced mothers or information on mothers cannot be distinguished from the study sample (e.g., parents, offspring, women) |
| Chrispin (1998) | Resilient adaptation of church-affiliated young Haitian immigrants: A search for protective resources (academic resilience, emotional resilience) | Not focused on forcibly displaced mothers or information on mothers cannot be distinguished from the study sample (e.g., parents, offspring, women) |
| Christiansen et al. (2022) | "Whether you like my skin or not, I am here": skilled racial minority migrant women's experiences of navigating career challenges in the White Icelandic labor market | Not focused on forcibly displaced mothers or information on mothers cannot be distinguished from the study sample (e.g., parents, offspring, women) |
| Christophe et al. (2020) | Latent Profiles of American and Ethnic-Racial Identity in Latinx Mothers and Adolescents: Links to Behavioral Practices and Cultural Values | No information on protective factors of psychological resilience or risk factors for psychological resilience in mothers |
| Chumky et al. (2023) | How Do Left-Behind Families Adapt to the Salinity-Induced Male Out-Migration Context? A Case Study of Shyamnagar Sub-District in Coastal Bangladesh | Not focused on forcibly displaced mothers or information on mothers cannot be distinguished from the study sample (e.g., parents, offspring, women) |
| Ciaramella et al. (2022) | Promotion of Resilience in Migrants: A Systematic Review of Study and Psychosocial Intervention | Review, commentary papers and research protocols (i.e., not reporting novel data/findings) |
| Clausing and Non (2021) | Epigenetics as a Mechanism of Developmental Embodiment of Stress, Resilience, and Cardiometabolic Risk Across Generations of Latinx Immigrant Families | Not focused on forcibly displaced mothers or information on mothers cannot be distinguished from the study sample (e.g., parents, offspring, women) |
| Clausing et al. (2021) | Epigenetic age associates with psychosocial stress and resilience in children of Latinx immigrants | Not focused on forcibly displaced mothers or information on mothers cannot be distinguished from the study sample (e.g., parents, offspring, women) |
| Clauss et al. (2023) | Development of a transdiagnostic, resilience-focused intervention for at-risk adolescents | Not focused on forcibly displaced mothers or information on mothers cannot be distinguished from the study sample (e.g., parents, offspring, women) |
| Coggins (2000) | Resilience and risk: Urban high school students share their perspectives | Not focused on forcibly displaced mothers or information on mothers cannot be distinguished from the study sample (e.g., parents, offspring, women) |
| Corley et al. (2021) | Exploring African Immigrant Women's Pre- and Post-Migration Exposures to Stress and Violence, Sources of Resilience, and Psychosocial Outcomes | Not focused on forcibly displaced mothers or information on mothers cannot be distinguished from the study sample (e.g., parents, offspring, women) |
| Cotton (2019) | Migration and young women's access to maternal healthcare in sub-Saharan Africa | No information on protective factors of psychological resilience or risk factors for psychological resilience in mothers |
| Cox (2017) | Promoting resilience with the Unidos Se Puede! Program: An example of translational research for Latino families | Not focused on forcibly displaced mothers or information on mothers cannot be distinguished from the study sample (e.g., parents, offspring, women) |
| Cupito (2018) | Cultural and contextual risk and resilience processes in the family stress model for Latino families | No information on protective factors of psychological resilience or risk factors for psychological resilience in mothers |
| Cyril et al. (2016) | Relationship between body mass index and family functioning, family communication, family type and parenting style among African migrant parents and children in Victoria, Australia: a parent-child dyad study | No information on protective factors of psychological resilience or risk factors for psychological resilience in mothers |
| Dalgaard et al. (2020) | Family violence in traumatized refugee families: A mixed methods study of mother/child dyadic functioning, parental symptom levels and children's psychosocial adjustment | No information on protective factors of psychological resilience or risk factors for psychological resilience in mothers |
| Davis et al. (2021) | A longitudinal study of paternal and maternal involvement and neighborhood risk on recent immigrant Latino/a youth prosocial behaviors | Not focused on forcibly displaced mothers or information on mothers cannot be distinguished from the study sample (e.g., parents, offspring, women) |
| de Torres et al. (2018) | Factors promoting resilience among Mexican immigrant women in the United States: Applying a positive deviance approach | Not focused on forcibly displaced mothers or information on mothers cannot be distinguished from the study sample (e.g., parents, offspring, women) |
| Denkinger et al. (2018) | Secondary Traumatization in Caregivers Working With Women and Children Who Suffered Extreme Violence by the "Islamic State" | Not focused on forcibly displaced mothers or information on mothers cannot be distinguished from the study sample (e.g., parents, offspring, women) |
| Denzongpa and Nichols (2020) | We Can't Step Back: Women Specially ... A Narrative Case Study on Resilience, Independence, and Leadership of a Bhutanese Refugee Woman | Not focused on forcibly displaced mothers or information on mothers cannot be distinguished from the study sample (e.g., parents, offspring, women) |
| Diaz (2004) | Maternal depressive symptomatology and marital quality among low SES Latina mothers: A longitudinal study | No information on protective factors of psychological resilience or risk factors for psychological resilience in mothers |
| Dunbar (2010) | Conditions of early maternal separation and later adult functioning in a Caribbean population | Not focused on forcibly displaced mothers or information on mothers cannot be distinguished from the study sample (e.g., parents, offspring, women) |
| Dybdahl et al. (2021) | How can we support parents and caregivers in reception centres and early phases of resettlement? | Not focused on forcibly displaced mothers or information on mothers cannot be distinguished from the study sample (e.g., parents, offspring, women) |
| East et al. (2018) | The Impact of Refugee Mothers' Trauma, Posttraumatic Stress, and Depression on Their Children's Adjustment | No information on protective factors of psychological resilience or risk factors for psychological resilience in mothers |
| Edberg et al. (2022) | The Adelante Project: Realities, Challenges and Successes in Addressing Health Disparities Among Central American Immigrant Youth | Not focused on forcibly displaced mothers or information on mothers cannot be distinguished from the study sample (e.g., parents, offspring, women) |
| Egilson et al. (2019) | Migrant families with disabled children: Parent perspectives | Not focused on forcibly displaced mothers or information on mothers cannot be distinguished from the study sample (e.g., parents, offspring, women) |
| El-Radi (2015) | The resettlement experiences of Southern Sudanese women refugees in Minnesota | Not focused on forcibly displaced mothers or information on mothers cannot be distinguished from the study sample (e.g., parents, offspring, women) |
| El-Sayed et al. (2014) | Trends in the Mexican infant mortality paradox over the past two decades | No information on protective factors of psychological resilience or risk factors for psychological resilience in mothers |
| Elizur and Perednik (2003) | Prevalence and description of selective mutism in immigrant and native families: A controlled study | No information on protective factors of psychological resilience or risk factors for psychological resilience in mothers |
| Ellis et al. (2023) | Supporting Women and Children Returning from Violent Extremist Contexts: Proposing a 5R Framework to Inform Program and Policy Development | Review, commentary papers and research protocols (i.e., not reporting novel data/findings) |
| Eltanamly et al. (2023) | Strengthening parental self-efficacy and resilience: A within-subject experimental study with refugee parents of adolescents | Not focused on forcibly displaced mothers or information on mothers cannot be distinguished from the study sample (e.g., parents, offspring, women) |
| Eo and Ji-Soo (2018) | Parenting stress and maternal‚Äìchild interactions among preschool mothers from the Philippines, Korea, and Vietnam: A cross-sectional, comparative study | No information on protective factors of psychological resilience or risk factors for psychological resilience in mothers |
| Erdemir (2022) | Home-Based Early Education for Refugee and Local Children via Mothers: A Model of Contextually Sensitive Early Intervention | No information on protective factors of psychological resilience or risk factors for psychological resilience in mothers |
| Farah (2007) | Spirituality among immigrant children adjusting to life challenges | No information on protective factors of psychological resilience or risk factors for psychological resilience in mothers |
| Farfan-Santos (2019) | Undocumented Motherhood: Gender, Maternal Identity, and the Politics of Health Care | No information on protective factors of psychological resilience or risk factors for psychological resilience in mothers |
| Ferrer-Wreder et al. (2021) | Exploring Lived Experiences of Parents of Youth and Youth with a Foreign Background in Sweden | Not focused on forcibly displaced mothers or information on mothers cannot be distinguished from the study sample (e.g., parents, offspring, women) |
| Gagnon and Stewart (2014) | Resilience in international migrant women following violence associated with pregnancy | Not focused on forcibly displaced mothers or information on mothers cannot be distinguished from the study sample (e.g., parents, offspring, women) |
| Gagnon et al. (2013) | Developing population interventions with migrant women for maternal-child health: a focused ethnography | Not focused on forcibly displaced mothers or information on mothers cannot be distinguished from the study sample (e.g., parents, offspring, women) |
| Galano et al. (2017) | Posttraumatic Stress Disorder in Latina Women: Examining the Efficacy of the Moms' Empowerment Program | No information on protective factors of psychological resilience or risk factors for psychological resilience in mothers |
| Gao et al. (2020) | An ecological investigation of resilience among rural-urban migrant adolescents of low socioeconomic status families in China | Not focused on forcibly displaced mothers or information on mothers cannot be distinguished from the study sample (e.g., parents, offspring, women) |
| Garcia (2019) | "No vamos a tapar el sol con un dedo": Maternal Communication Concerning Immigration Status | No information on protective factors of psychological resilience or risk factors for psychological resilience in mothers |
| Gehlen et al. (2023) | Vulnerabilities of Venezuelan refugee women: violence and intersectional social relations | Not focused on forcibly displaced mothers or information on mothers cannot be distinguished from the study sample (e.g., parents, offspring, women) |
| Georgis (2014) | More than meets the eye: Immigrant and refugee adjustment, education, and acculturation in Canada | No information on protective factors of psychological resilience or risk factors for psychological resilience in mothers |
| German (2008) | Educational psychologists promoting the emotional wellbeing and resilience of refugee parents | Review, commentary papers and research protocols (i.e., not reporting novel data/findings) |
| Gerrard et al. (2023) | How to help a child through couple relationship strengthening | Review, commentary papers and research protocols (i.e., not reporting novel data/findings) |
| Gibson (2016) | Expressions of resilience in at-risk women | Not focused on forcibly displaced mothers or information on mothers cannot be distinguished from the study sample (e.g., parents, offspring, women) |
| Ginesini (2018) | Forced migration: Trauma, faith, and resilience | Not focused on forcibly displaced mothers or information on mothers cannot be distinguished from the study sample (e.g., parents, offspring, women) |
| Giordano et al. (2014) | Risk and protection in mental health among syrian children displaced in lebanon | Not focused on forcibly displaced mothers or information on mothers cannot be distinguished from the study sample (e.g., parents, offspring, women) |
| Gonçalves and Matos (2020) | Mental health of multiple victimized immigrant women in Portugal: Does resilience make a difference? | Not focused on forcibly displaced mothers or information on mothers cannot be distinguished from the study sample (e.g., parents, offspring, women) |
| Gordillo et al. (2020) | Mothers' adjustment to autism: Exploring the roles of autism knowledge and culture | No information on protective factors of psychological resilience or risk factors for psychological resilience in mothers |
| Graham (2013) | Child Health and Migrant Parents in South-East Asia: Risk and Resilience among Primary School-Aged Children | No information on protective factors of psychological resilience or risk factors for psychological resilience in mothers |
| Gredebäck (2022) | Fluid intelligence in refugee children. A cross-sectional study of potential risk and resilience factors among Syrian refugee children and their parents | Not focused on forcibly displaced mothers or information on mothers cannot be distinguished from the study sample (e.g., parents, offspring, women) |
| Greer et al. (2013) | The Cultural Voice of Immigrant Latina Women and the Meaning of Femininity: A Phenomenological Study | No information on protective factors of psychological resilience or risk factors for psychological resilience in mothers |
| Guan et al. (2023) | Negative perceived context of reception and sociocultural resources on mental health among Hispanic and Somali adolescents | Not focused on forcibly displaced mothers or information on mothers cannot be distinguished from the study sample (e.g., parents, offspring, women) |
| Guerra et al. (2023) | Intergroup relations, acculturation orientations, and adaptation of Turkish immigrant descent parents across Europe | Not focused on forcibly displaced mothers or information on mothers cannot be distinguished from the study sample (e.g., parents, offspring, women) |
| Guntzviller et al (2019) | Mother-Adolescent Communication in Low-Income, Latino Families during Language Brokering: Examining the Theory of Resilience and Relational Load | Not focused on forcibly displaced mothers or information on mothers cannot be distinguished from the study sample (e.g., parents, offspring, women) |
| Gupta et al. (2023) | Ea$ing into the USA: study protocol for adapting the Economic and Social Empowerment (EA$E) intervention for US-based, forcibly based populations | Review, commentary papers and research protocols (i.e., not reporting novel data/findings) |
| Haar et al. (2020) | Strong families: a new family skills training programme for challenged and humanitarian settings: a single-arm intervention tested in Afghanistan | No information on protective factors of psychological resilience or risk factors for psychological resilience in mothers |
| Hainmueller et al. (2017) | Protecting unauthorized immigrant mothers improves their children's mental health | No information on protective factors of psychological resilience or risk factors for psychological resilience in mothers |
| Han (2013) | A qualitative study examining influences on identity development for biracial adolescents and emerging adults | No information on protective factors of psychological resilience or risk factors for psychological resilience in mothers |
| Harden et al. (2021) | Intimate Partner Violence, Parenting, and Toddler Behavior among Low-Income Latinx Families | Not focused on forcibly displaced mothers or information on mothers cannot be distinguished from the study sample (e.g., parents, offspring, women) |
| Hashimoto-Govindasamy and Rose (2011) | An ethnographic process evaluation of a community support program with Sudanese refugee women in western Sydney | Not focused on forcibly displaced mothers or information on mothers cannot be distinguished from the study sample (e.g., parents, offspring, women) |
| Heilemann et al. (2002) | Strengths and vulnerabilities of women of Mexican descent in relation to depressive symptoms | Not focused on forcibly displaced mothers or information on mothers cannot be distinguished from the study sample (e.g., parents, offspring, women) |
| Heilemann et al. (2011) | Schema therapy, motivational interviewing, and collaborative-mapping as treatment for depression among low income, second generation Latinas | Not focused on forcibly displaced mothers or information on mothers cannot be distinguished from the study sample (e.g., parents, offspring, women) |
| Heller et al. (2022) | Complications and Healthcare Consumption of Pregnant Women with a Migrant Background: Could There be an Association with Psychological Distress? | No information on protective factors of psychological resilience or risk factors for psychological resilience in mothers |
| Hennegan et al. (2015) | Another country, another language and a new baby: A quantitative study of the postnatal experiences of migrant women in Australia | No information on protective factors of psychological resilience or risk factors for psychological resilience in mothers |
| Herrero-Arias et al. (2020) | The more you go to the mountains, the better parent you are'. Migrant parents in Norway navigating risk discourses in professional advice on family leisure and outdoor play | No information on protective factors of psychological resilience or risk factors for psychological resilience in mothers |
| Hillary et al. (2022) | When the World Turns Upside Down, Live Like a Bat!' Idioms of Suffering, Coping, and Resilience among Elderly Female Zande Refugees in Kiryandongo Refugee Settlement, Uganda (2019-20) | Review, commentary papers and research protocols (i.e., not reporting novel data/findings) |
| Hoffman et al. (2019) | Intergenerational effects of trauma resulting from torture and war: Associations between maternal caregivers' mental health and youth psychosocial adjustment | No information on protective factors of psychological resilience or risk factors for psychological resilience in mothers |
| Hoffman et al. (2020) | Mechanisms Explaining the Relationship Between Maternal Torture Exposure and Youth Adjustment In Resettled Refugees: A Pilot Examination of Generational Trauma Through Moderated Mediation | No information on protective factors of psychological resilience or risk factors for psychological resilience in mothers |
| Höltge et al. (2021) | A cross-country network analysis of adolescent resilience | Not focused on forcibly displaced mothers or information on mothers cannot be distinguished from the study sample (e.g., parents, offspring, women) |
| Hong et al. (2021) | Stress among Korean immigrant parents of children with diagnosed needs amid the COVID-19 pandemic | Not focused on forcibly displaced mothers or information on mothers cannot be distinguished from the study sample (e.g., parents, offspring, women) |
| Hong et al. (2021) | Parental stress of Korean immigrants in the US: Meeting Child and Youth's educational needs amid the COVID-19 pandemic | Not focused on forcibly displaced mothers or information on mothers cannot be distinguished from the study sample (e.g., parents, offspring, women) |
| Honig et al. (1997) | Child resilience in Taiwanese immigrant families as a function of maternal supports and maternal employment | No information on protective factors of psychological resilience or risk factors for psychological resilience in mothers |
| Hosin (2001) | Children of traumatized and exiled refugee families: resilience and vulnerability. A case study report | Review, commentary papers and research protocols (i.e., not reporting novel data/findings) |
| Hosin et al. (2006) | The Relationship Between Psychological Well-Being and Adjustment of Both Parents and Children of Exiled and Traumatized Iraqi Refugees | No information on protective factors of psychological resilience or risk factors for psychological resilience in mothers |
| Huang et al. (2017) | Cultural adaptation, parenting and child mental health among English speaking Asian American immigrant families | No information on protective factors of psychological resilience or risk factors for psychological resilience in mothers |
| Huang et al. (2019) | Family socioeconomic status and emotional adaptation among rural-to-urban migrant adolescents in China: The moderating roles of adolescent's resilience and parental positive emotion | No information on protective factors of psychological resilience or risk factors for psychological resilience in mothers |
| Huntley et al. (2021) | The Parenting Process in Syrian Refugees: A Grounded Theory Study | Not focused on forcibly displaced mothers or information on mothers cannot be distinguished from the study sample (e.g., parents, offspring, women) |
| Igreja (2003) | The effects of traumatic experiences on the infant-mother relationship in the former war zones of central Mozambique: The case of Madzawde in Gorongosa | No information on protective factors of psychological resilience or risk factors for psychological resilience in mothers |
| Im (2021) | Falling Through the Cracks: Stress and Coping in Migration and Resettlement Among Marginalized Hmong Refugee Families in the United States | Not focused on forcibly displaced mothers or information on mothers cannot be distinguished from the study sample (e.g., parents, offspring, women) |
| Islam et al. (2018) | Trauma, migration and mental health in a sample of asylum-seeking women in Italy | Not focused on forcibly displaced mothers or information on mothers cannot be distinguished from the study sample (e.g., parents, offspring, women) |
| Izzo (1999) | Predictors of parenting practices and children's adjustment in Mexican immigrant families | Not focused on forcibly displaced mothers or information on mothers cannot be distinguished from the study sample (e.g., parents, offspring, women) |
| Izzo and Weiss (2000) | Parental self-efficacy and social support as predictors of parenting practices and children's socioemotional adjustment in Mexican immigrant families | No information on protective factors of psychological resilience or risk factors for psychological resilience in mothers |
| Jefferis and Theron (2018) | Explanations of resilience in women and girls: How applicable to black South African girls | Review, commentary papers and research protocols (i.e., not reporting novel data/findings) |
| Ji (2007) | Maternal mental health, education, acculturation, and social support as predictors of the parenting of Asian American and Asian immigrant mothers | No information on protective factors of psychological resilience or risk factors for psychological resilience in mothers |
| Jo (2020) | The significance of resilience in mental health promotion of marriage immigrant women: A qualitative study of factors and processes | Not focused on forcibly displaced mothers or information on mothers cannot be distinguished from the study sample (e.g., parents, offspring, women) |
| Jolof et al. (2022) | Experiences of armed conflicts and forced migration among women from countries in the Middle East, Balkans, and Africa: a systematic review of qualitative studies | Review, commentary papers and research protocols (i.e., not reporting novel data/findings) |
| Ju et al. (2023) | Resilience and (Dis)empowerment: Use of Social Media Among Female Mainland Low-Skilled Workers in Macao During the COVID-19 Pandemic | Not focused on forcibly displaced mothers or information on mothers cannot be distinguished from the study sample (e.g., parents, offspring, women) |
| Juang (2018) | Reactive and Proactive Ethnic-Racial Socialization Practices of Second-Generation Asian American Parents | No information on protective factors of psychological resilience or risk factors for psychological resilience in mothers |
| Jung (2012) | Family Functioning and Early Learning Practices in Immigrant Homes | Not focused on forcibly displaced mothers or information on mothers cannot be distinguished from the study sample (e.g., parents, offspring, women) |
| Kabakian-Khasholian et al. (2022) | "A person who does not have money does not enter": a qualitative study on refugee women's experiences of respectful maternity care | No information on protective factors of psychological resilience or risk factors for psychological resilience in mothers |
| Kaiser et al. (2015) | Adaptation of a Culturally Relevant Nutrition and Physical Activity Program for Low-Income, Mexican-Origin Parents With Young Children | No information on protective factors of psychological resilience or risk factors for psychological resilience in mothers |
| Kaufmann et al. (2022) | Maternal mental healthcare needs of refugee women in a State Registration and Reception Centre in Germany: A descriptive study | No information on protective factors of psychological resilience or risk factors for psychological resilience in mothers |
| Kentoffio et al. (2015) | Maternal health services use: Comparing refugee, immigrant and native populations | Not focused on forcibly displaced mothers or information on mothers cannot be distinguished from the study sample (e.g., parents, offspring, women) |
| Kentoffio et al. (2016) | Use of maternal health services: comparing refugee, immigrant and US-born populations | No information on protective factors of psychological resilience or risk factors for psychological resilience in mothers |
| Khalil et al. (2022) | Acculturative Stress and Postpartum Depressive Symptoms among Immigrant Arab American Couples | Not focused on forcibly displaced mothers or information on mothers cannot be distinguished from the study sample (e.g., parents, offspring, women) |
| Khan (2022) | 1.5 A Review of Two Studies on Immigrant and Refugee Youths' Mental Health in Canada: Implications for Policy and Practice | No information on protective factors of psychological resilience or risk factors for psychological resilience in mothers |
| Khawaja et al. (2021) | Building resilience in transcultural adults: investigating the effect of a strength-based programme | Not focused on forcibly displaced mothers or information on mothers cannot be distinguished from the study sample (e.g., parents, offspring, women) |
| Khawaja et al. (2023) | Building resilience in transcultural adults (BRiTA): Developing a novel preventative intervention | Not focused on forcibly displaced mothers or information on mothers cannot be distinguished from the study sample (e.g., parents, offspring, women) |
| Kikhia et al. (2021) | Exploring how Syrian women manage their health after migration to Germany: results of a qualitative study | Not focused on forcibly displaced mothers or information on mothers cannot be distinguished from the study sample (e.g., parents, offspring, women) |
| Kim (2018) | Social support, acculturation stress, and parenting stress among marriage-migrant women | No information on protective factors of psychological resilience or risk factors for psychological resilience in mothers |
| Kim and Grant (1997) | Immigration Patterns, Social Support, and Adaptation Among Korean Immigrant Women and Korean American Women | Not focused on forcibly displaced mothers or information on mothers cannot be distinguished from the study sample (e.g., parents, offspring, women) |
| Kim et al. (2014) | Maternal Acculturation Process of Married Immigrant Women in Korea | Not focused on forcibly displaced mothers or information on mothers cannot be distinguished from the study sample (e.g., parents, offspring, women) |
| Kindermann et al. (2017) | Prevalence of and Risk Factors for Secondary Traumatization in Interpreters for Refugees: A Cross-Sectional Study | Not focused on forcibly displaced mothers or information on mothers cannot be distinguished from the study sample (e.g., parents, offspring, women) |
| Kishinvevsky (2001) | Survival in the land of glamor: The experience of three generations of women who emigrated from the former Soviet Union (acculturation in the United States and its influence on their perceptions and lifestyles) | No information on protective factors of psychological resilience or risk factors for psychological resilience in mothers |
| Kong et al. (2021) | Perceived social support, resilience and health self-efficacy among migrant older adults: A moderated mediation analysis | Not focused on forcibly displaced mothers or information on mothers cannot be distinguished from the study sample (e.g., parents, offspring, women) |
| Kumar et al. (2017) | Promoting children's resilience by strengthening parenting practices in families under extreme stress: The Parent Management Training-Oregon model | Review, commentary papers and research protocols (i.e., not reporting novel data/findings) |
| Kuru et al. (2021) | Refugee Children's Resilience: A Qualitative Social Ecological Study of Life in a Camp | No information on protective factors of psychological resilience or risk factors for psychological resilience in mothers |
| Lakkis et al. (2020) | A Pilot Intervention to Promote Positive Parenting in Refugees from Syria in Lebanon and Jordan | No information on protective factors of psychological resilience or risk factors for psychological resilience in mothers |
| Lau et al. (2011) | Group Parent Training With Immigrant Chinese Families: Enhancing Engagement and Augmenting Skills Training | No information on protective factors of psychological resilience or risk factors for psychological resilience in mothers |
| Lee (2007) | Temporal changes in immigration stress, adaptation, and the factors contributing to the immigration-adaptation process in newly immigrant parents | Not focused on forcibly displaced mothers or information on mothers cannot be distinguished from the study sample (e.g., parents, offspring, women) |
| Lee (2023) | Knowledge, confidence, and educational needs of newborn care among North Korean refugee women: a descriptive study | No information on protective factors of psychological resilience or risk factors for psychological resilience in mothers |
| Lee et al. (2018) | The Role of Ethnic Socialization, Ethnic Identity and Self-Esteem: Implications for Bi-ethnic Adolescents' School Adjustment | Not focused on forcibly displaced mothers or information on mothers cannot be distinguished from the study sample (e.g., parents, offspring, women) |
| Lee et al. (2022) | Influence of hardiness, mother-child interactions, and social support on parenting stress among North Korean refugee mothers: a cross-sectional study | No information on protective factors of psychological resilience or risk factors for psychological resilience in mothers |
| Leidy et al. (2010) | Positive Parenting, Family Cohesion, and Child Social Competence Among Immigrant Latino Families | No information on protective factors of psychological resilience or risk factors for psychological resilience in mothers |
| Lemus-Way | Strengths and Resilience of Migrant Women in Transit: an Analysis of the Narratives of Central American Women in Irregular Transit Through Mexico Towards the USA | Not focused on forcibly displaced mothers or information on mothers cannot be distinguished from the study sample (e.g., parents, offspring, women) |
| Lenderts et al. (2021) | The Role of Culture in Shaping Health Perceptions and Behaviors of Resettled Karen Refugees | No information on protective factors of psychological resilience or risk factors for psychological resilience in mothers |
| Lieberman et al. (2011) | Correlates of maternal interactive behavior in latina immigrant mothers of 1-year old infants | No information on protective factors of psychological resilience or risk factors for psychological resilience in mothers |
| Lim (2021) | Relationship between Marriage Immigrant Mothers' Acculturative Stress and Their Adolescent Children's Career Decidedness in South Korea: Mediating Roles of Parenting and School Adjustment | No information on protective factors of psychological resilience or risk factors for psychological resilience in mothers |
| Linden et al. (2023) | Raising teenage children in disadvantaged neighbourhoods: the experiences and challenges of immigrant mothers in Sweden | No information on protective factors of psychological resilience or risk factors for psychological resilience in mothers |
| Liu et al. (2020) | Strengths-based inquiry of resiliency factors among refugees in Metro Vancouver: A comparison of newly-arrived and settled refugees | Not focused on forcibly displaced mothers or information on mothers cannot be distinguished from the study sample (e.g., parents, offspring, women) |
| Lorén-Guerrero et al. (2018) | Assessment of significant psychological distress at the end of pregnancy and associated factors | Not focused on forcibly displaced mothers or information on mothers cannot be distinguished from the study sample (e.g., parents, offspring, women) |
| Lu et al. (2023) | How does parental emotion regulation influence chinese migrant children‚Äôs resilience? The chain mediating role of self-efficacy and hope | No information on protective factors of psychological resilience or risk factors for psychological resilience in mothers |
| MacDonnell et al. (2012) | Becoming Resilient: Promoting the Mental Health and Well-Being of Immigrant Women in a Canadian Context | Not focused on forcibly displaced mothers or information on mothers cannot be distinguished from the study sample (e.g., parents, offspring, women) |
| Mak et al. (2021) | Intergenerational Transmission of Traumatic Stress and Resilience Among Cambodian Immigrant Families Along Coastal Alabama: Family Narratives | Not focused on forcibly displaced mothers or information on mothers cannot be distinguished from the study sample (e.g., parents, offspring, women) |
| Mangrio et al. (2020) | Newly arrived refugee parents in Sweden and their experience of the resettlement process: A qualitative study | Not focused on forcibly displaced mothers or information on mothers cannot be distinguished from the study sample (e.g., parents, offspring, women) |
| Marcovitch et al. (1995) | ROMANIAN ADOPTION - PARENTS DREAMS, NIGHTMARES, AND REALITIES | No information on protective factors of psychological resilience or risk factors for psychological resilience in mothers |
| Marsiglia et al. (2011) | Hopelessness, family stress, and depression among Mexican-heritage mothers in the Southwest | No information on protective factors of psychological resilience or risk factors for psychological resilience in mothers |
| Martinez et al. (2011) | Time in US Residency and the Social, Behavioral, and Emotional Adjustment of Latino Immigrant Families | No information on protective factors of psychological resilience or risk factors for psychological resilience in mothers |
| Martinez-Taboada et al. (2017) | Positive affective prediction as a factor of social emotional protection in transnational mothers before and after family reunification: relationship with life satisfaction, emotional regulation, social loneliness, resilience and stress | No information on protective factors of psychological resilience or risk factors for psychological resilience in mothers |
| Martinez-Taboada et al. (2017) | La predicción afectiva positiva como factor de protección socio- emocional en madres transnacionales antes y después de la reagrupación familiar: relación con la satisfacción con la vida, regulación emocional, soledad social, resiliencia y estrés = Positive affective prediction as a factor of social emotional protection in transnational mothers before and after family reunification: Relationship with life satisfaction, emotional regulation, social loneliness, resilience and stress | duplicate |
| Maru et al. (2023) | The protective effects of social support and family functioning on parenting stress among Hispanic/Latino/a American immigrant parents with traumatic life experiences: A mediation analysis | No information on protective factors of psychological resilience or risk factors for psychological resilience in mothers |
| Mayer et al. (2020) | Relations between traumatic life events and mental health of Eritrean asylum-seeking mothers and their children's mental health | No information on protective factors of psychological resilience or risk factors for psychological resilience in mothers |
| Mazar (2015) | Birth across borders: A comparative study of Guatemalan-Maya maternal care in San Miguel Acatan and Palm Beach County | Not focused on forcibly displaced mothers or information on mothers cannot be distinguished from the study sample (e.g., parents, offspring, women) |
| McCloskey et al. (1995) | The psychological effects of political and domestic violence on central-American and Mexican immigrant mothers and children | No information on protective factors of psychological resilience or risk factors for psychological resilience in mothers |
| McConnell et al. (2021) | Vulnerable and Resilient: Legal Status, Sources of Support, Maternal Knowledge, and the Family Routines of Mexican and Central American-origin Mothers in Los Angeles | No information on protective factors of psychological resilience or risk factors for psychological resilience in mothers |
| McMahon et al. (2020) | Child Persecutor or Child Savior? The Impact of Trauma and Migration on a Mother's Ambivalence in Relation to her Child | No information on protective factors of psychological resilience or risk factors for psychological resilience in mothers |
| McNaughton et al. (2014) | Adaptation and Feasibility of a Communication Intervention for Mexican Immigrant Mothers and Children in a School Setting | No information on protective factors of psychological resilience or risk factors for psychological resilience in mothers |
| Miao et al. (2018) | Spillover of stress to Chinese Canadian immigrants' parenting: Impact of acculturation and parent–child stressors | No information on protective factors of psychological resilience or risk factors for psychological resilience in mothers |
| Michel (2017) | The immigrant mother: A phenomenological study of Latina maternal attachment | No information on protective factors of psychological resilience or risk factors for psychological resilience in mothers |
| Miszkurka et al. (2010) | Contributions of Immigration to Depressive Symptoms Among Pregnant Women in Canada | No information on protective factors of psychological resilience or risk factors for psychological resilience in mothers |
| Montoya-Williams et al. (2021) | Resilience During Pregnancy by Race, Ethnicity and Nativity: Evidence of a Hispanic Immigrant Advantage | No information on protective factors of psychological resilience or risk factors for psychological resilience in mothers |
| Morales (2023) | Strengths and Resilience Among Central American Parents Seeking Asylum in the United States | Not focused on forcibly displaced mothers or information on mothers cannot be distinguished from the study sample (e.g., parents, offspring, women) |
| Moran et al. (2017) | Hoping for a better tomorrow: a qualitative study of stressors, informal social support and parental coping in a Direct Provision centre in the West of Ireland | Not focused on forcibly displaced mothers or information on mothers cannot be distinguished from the study sample (e.g., parents, offspring, women) |
| Mortantz (2012) | The Divergent Experiences of Children and Adults in the Relocation Process: Perspectives of Child and Parent Refugee Claimants in Montreal | No information on protective factors of psychological resilience or risk factors for psychological resilience in mothers |
| Muong (2009) | The effects of parental unresolved trauma on second generation Cambodian adolescents | No information on protective factors of psychological resilience or risk factors for psychological resilience in mothers |
| Mussino et al. (2012) | The fertility of immigrants after arrival: The Italian case | No information on protective factors of psychological resilience or risk factors for psychological resilience in mothers |
| Mussino et al. (2015) | Motherhood of foreign women in Lombardy: Testing the effects of migration by citizenship | No information on protective factors of psychological resilience or risk factors for psychological resilience in mothers |
| Mussino et al. (2019) | Transition to third birth among immigrant mothers in Sweden: Does having two daughters accelerate the process? | No information on protective factors of psychological resilience or risk factors for psychological resilience in mothers |
| Ng et al. (2017) | Immigrant Chinese Mothers' Socialization of Achievement in Children: A Strategic Adaptation to the Host Society | No information on protective factors of psychological resilience or risk factors for psychological resilience in mothers |
| Niscanci (2020) | Parental monitoring in Turkish immigrant families in the United States | No information on protective factors of psychological resilience or risk factors for psychological resilience in mothers |
| Noori et al. (2016) | Pregnancy outcomes in non-UK born women in a tertiary level maternity unit: A pilot study | No information on protective factors of psychological resilience or risk factors for psychological resilience in mothers |
| Nunes et al. (2021) | Parents' perceived social support and children's psychological adjustment | No information on protective factors of psychological resilience or risk factors for psychological resilience in mothers |
| O'Mahony (2011) | Immigrant and refugee women's voices: Exploring postpartum depression help seeking experiences and access to mental health care | No information on protective factors of psychological resilience or risk factors for psychological resilience in mothers |
| Ögtem-Young et al. (2018) | Faith Resilience: Everyday Experiences | Review, commentary papers and research protocols (i.e., not reporting novel data/findings) |
| Olson et al. (2019) | Recent Canadian efforts to develop population-level pregnancy intervention studies to mitigate effects of natural disasters and other tragedies | Review, commentary papers and research protocols (i.e., not reporting novel data/findings) |
| Onofre Vasquez (2018) | Latino parents in a primarily White and relatively affluent school district: The story of their engagement in their children's school | Not focused on forcibly displaced mothers or information on mothers cannot be distinguished from the study sample (e.g., parents, offspring, women) |
| Ornelas et al. (2009) | Challenges and Strategies to Maintaining Emotional Health Qualitative Perspectives of Mexican Immigrant Mothers | Not focused on forcibly displaced mothers or information on mothers cannot be distinguished from the study sample (e.g., parents, offspring, women) |
| Osman et al. (2022) | The implementation of a culturally tailored parenting support programme for Somali immigrant parents living in Sweden. A process evaluation | No information on protective factors of psychological resilience or risk factors for psychological resilience in mothers |
| Osornio et al. (2022) | Developmental trajectories of internalizing distress among ethnic minoritized mothers following childbirth: Associations with early child psychological adjustment | No information on protective factors of psychological resilience or risk factors for psychological resilience in mothers |
| Pangas et al. (2018) | Refugee women's experiences of motherhood and maternity services in a new country: A meta-ethnographic study | No information on protective factors of psychological resilience or risk factors for psychological resilience in mothers |
| Panter-Brick et al. (2014) | Caregiver-child mental health: a prospective study in conflict and refugee settings | No information on protective factors of psychological resilience or risk factors for psychological resilience in mothers |
| Paris (2008) | "For the Dream of Being Here, One Sacrifices ...": Voices of Immigrant Mothers in a Home Visiting Program | No information on protective factors of psychological resilience or risk factors for psychological resilience in mothers |
| Parmar et al. (2023) | Latina mothers' perspectives on adverse experiences and protection of Latinx youth in an agricultural community | No information on protective factors of psychological resilience or risk factors for psychological resilience in mothers |
| Parra-Cardona et al. (2017) | Examining the Impact of Differential Cultural Adaptation With Latina/o Immigrants Exposed to Adapted Parent Training Interventions | No information on protective factors of psychological resilience or risk factors for psychological resilience in mothers |
| Pejic (2017) | Promoting healthy family transition and support in Somali refugee parents: Outcomes of a community-based program | No information on protective factors of psychological resilience or risk factors for psychological resilience in mothers |
| Perera et al. (2022) | Access to community-based reproductive health services and incidence of low birthweight delivery among refugee and displaced mothers: a retrospective study in the Thailand-Myanmar border region | No information on protective factors of psychological resilience or risk factors for psychological resilience in mothers |
| Pezerovic et al. (2019) | Parents in Exile: Challenges of Parenting among Refugees and Asylum Seekers in Bulgaria | Not focused on forcibly displaced mothers or information on mothers cannot be distinguished from the study sample (e.g., parents, offspring, women) |
| Phipps (2022) | Thematic Analysis of Intergenerational Transmission of Trauma in Latinx Immigrant Families in the Southern US | No information on protective factors of psychological resilience or risk factors for psychological resilience in mothers |
| Platt (2021) | 31.3 Group well-child care model for Latino children in immigrant families: adapting to and learning from the Covid-19 context | Not focused on forcibly displaced mothers or information on mothers cannot be distinguished from the study sample (e.g., parents, offspring, women) |
| Poeran et al. (2013) | Social deprivation and adverse perinatal outcomes among Western and non-Western pregnant women in a Dutch urban population | No information on protective factors of psychological resilience or risk factors for psychological resilience in mothers |
| Qu et al. (2021) | Dyadic effects of fluid mindset on psychological growth in immigrant mothers and their children: Indirect effect of resilience | Not focused on forcibly displaced mothers or information on mothers cannot be distinguished from the study sample (e.g., parents, offspring, women) |
| Qu et al. (2023) | Variations in adaptation profiles among Chinese immigrant mothers and their children: A dyadic latent profile analysis | No information on protective factors of psychological resilience or risk factors for psychological resilience in mothers |
| Raffaelli et al. (2012) | Risk and Resilience in Rural Communities: The Experiences of Immigrant Latina Mothers | Not focused on forcibly displaced mothers or information on mothers cannot be distinguished from the study sample (e.g., parents, offspring, women) |
| Rania et al. (2018) | Parental Competence in Italy: A Comparison Between Italian and Immigrant Parents | No information on protective factors of psychological resilience or risk factors for psychological resilience in mothers |
| Ratnamohan et al. (2023) | Breaching the family walls: Modelling the impact of prolonged visa insecurity on asylum-seeking children | No information on protective factors of psychological resilience or risk factors for psychological resilience in mothers |
| Remennick (2005) | Immigration, gender, and psychosocial adjustment: A study of 150 immigrant couples in Israel | Not focused on forcibly displaced mothers or information on mothers cannot be distinguished from the study sample (e.g., parents, offspring, women) |
| Ren et al. (2020) | Maternal Attribution and Chinese Immigrant Children's Social Skills: The Mediating Role of Authoritative Parenting Practices | No information on protective factors of psychological resilience or risk factors for psychological resilience in mothers |
| Ren et al. (2021) | A person-centered examination of acculturation and psychological functioning among Chinese and Korean immigrant mothers in the United States | No information on protective factors of psychological resilience or risk factors for psychological resilience in mothers |
| Riccio (2014) | Dual Vulnerability of Being Both a Teen and an Immigrant Parent: Illustrations from an Italian Context | No information on protective factors of psychological resilience or risk factors for psychological resilience in mothers |
| Ritblatt (2023) | From trauma to resiliency: Trauma-informed practices for working with children, families, schools, and communities | Review, commentary papers and research protocols (i.e., not reporting novel data/findings) |
| Ritblatt et al. (2023) | Refugees' resettlement and traumatic experiences: Utilizing trauma-informed practices with refugee women to address war trauma and enhance resilience | Review, commentary papers and research protocols (i.e., not reporting novel data/findings) |
| Ritsner et al. (1993) | Psychological adjustment and distress among Soviet immigrant physicians: Demographic and background variables | Not focused on forcibly displaced mothers or information on mothers cannot be distinguished from the study sample (e.g., parents, offspring, women) |
| Rodriguez et al. (2022) | The effects of migration on parenting: voices of Latin American families in the Canary Islands | Not focused on forcibly displaced mothers or information on mothers cannot be distinguished from the study sample (e.g., parents, offspring, women) |
| Rose-Clarke et al. (2022) | Psychosocial resilience among left-behind adolescents in rural Thailand: A qualitative exploration | Not focused on forcibly displaced mothers or information on mothers cannot be distinguished from the study sample (e.g., parents, offspring, women) |
| Rosenberg et al. (2022) | Recently-Arrived Afghan Refugee Parents' Perspectives About Parenting, Education and Pediatric Medical and Mental Health Care Services | No information on protective factors of psychological resilience or risk factors for psychological resilience in mothers |
| Saasa et al. (2021) | Financial hardship, neighborhood cohesion and child externalizing behaviors: An extension of the family stress model among immigrant mothers | No information on protective factors of psychological resilience or risk factors for psychological resilience in mothers |
| Sabatier and Berry (2008) | The role of family acculturation, parental style, and perceived discrimination in the adaptation of second-generation immigrant youth in France and Canada | No information on protective factors of psychological resilience or risk factors for psychological resilience in mothers |
| Sami et al. (2019) | Giving birth in Switzerland: a qualitative study exploring migrant women's experiences during pregnancy and childbirth in Geneva and Zurich using focus groups | Not focused on forcibly displaced mothers or information on mothers cannot be distinguished from the study sample (e.g., parents, offspring, women) |
| Sanagavarapu (2012) | Does culture intersect with gender of the child in mothers' guidance of preschoolers' puzzle solving? | No information on protective factors of psychological resilience or risk factors for psychological resilience in mothers |
| Sanchez (2019) | Immigrant family reunification among central American undocumented minors in the US: Implications for counseling | No information on protective factors of psychological resilience or risk factors for psychological resilience in mothers |
| Saula et al. (2017) | Maternal care mechanisms in two comparative contexts: Mexico-Beijing | No information on protective factors of psychological resilience or risk factors for psychological resilience in mothers |
| Scharpf et al. (2019) | Prevalence and co-existence of morbidity of posttraumatic stress and functional impairment among Burundian refugee children and their parents | No information on protective factors of psychological resilience or risk factors for psychological resilience in mothers |
| Scharpf et al. (2021) | A socio-ecological analysis of risk, protective and promotive factors for the mental health of Burundian refugee children living in refugee camps | No information on protective factors of psychological resilience or risk factors for psychological resilience in mothers |
| Schoenborn et al. (2021) | Measuring the invisible: perinatal health outcomes of unregistered women giving birth in Belgium, a population-based study | No information on protective factors of psychological resilience or risk factors for psychological resilience in mothers |
| Sedighdeilami (2004) | Psychological adjustment of Iranian immigrants and refugees in Toronto (Ontario) | Not focused on forcibly displaced mothers or information on mothers cannot be distinguished from the study sample (e.g., parents, offspring, women) |
| Seo et al. (2023) | Longitudinal Relations Among Child Temperament, Parenting, and Acculturation in Predicting Korean American Children's Externalizing Problems | No information on protective factors of psychological resilience or risk factors for psychological resilience in mothers |
| Sharapova and Goguikian (2018) | Psychosocial and sociocultural factors influencing antenatal anxiety and depression in non-precarious migrant women | No information on protective factors of psychological resilience or risk factors for psychological resilience in mothers |
| Shaw et al. (2021) | Parenting Among Rohingya and Afghan Refugee Parents Residing in Malaysia | No information on protective factors of psychological resilience or risk factors for psychological resilience in mothers |
| Shin (2019) | How do mothers manage their privacy with adolescents? Exploring mother-adolescent communication in Mexican immigrant families | No information on protective factors of psychological resilience or risk factors for psychological resilience in mothers |
| Siegel (2022) | Examining depression and social and emotional development outcomes in parents and children in migrant and seasonal farmworker families | No information on protective factors of psychological resilience or risk factors for psychological resilience in mothers |
| Sigmarsdóttir et al. (2023) | Strengthening parenting among refugees in Europe (SPARE): initial feasibility in Iceland and Norway | Not focused on forcibly displaced mothers or information on mothers cannot be distinguished from the study sample (e.g., parents, offspring, women) |
| Silberman et al. (2020) | Friend Support and the Parenting of Latina Adolescent Mothers: The Moderating Role of Maternal Age | No information on protective factors of psychological resilience or risk factors for psychological resilience in mothers |
| Sim et al. (2018) | Modeling the effects of war exposure and daily stressors on maternal mental health, parenting, and child psychosocial adjustment: a cross-sectional study with Syrian refugees in Lebanon | No information on protective factors of psychological resilience or risk factors for psychological resilience in mothers |
| Sim et al. (2018) | Pathways linking war and displacement to parenting and child adjustment: A qualitative study with Syrian refugees in Lebanon | No information on protective factors of psychological resilience or risk factors for psychological resilience in mothers |
| Sim et al. (2021) | Acceptability and Preliminary Outcomes of a Parenting Intervention for Syrian Refugees | No information on protective factors of psychological resilience or risk factors for psychological resilience in mothers |
| Sim et al. (2023) | Resettlement, mental health, and coping: a mixed methods survey with recently resettled refugee parents in Canada | Not focused on forcibly displaced mothers or information on mothers cannot be distinguished from the study sample (e.g., parents, offspring, women) |
| Son (2018) | Established multicultural families' work and life: The impact of employment and perceived Korean husbands' practical support on migrant wives' life satisfaction | No information on protective factors of psychological resilience or risk factors for psychological resilience in mothers |
| Song et al. (2022) | Effects of Maternal Adjustment Enhancement Program Using Mobile-Based Education for Chinese Immigrant Women in Korea: A Quasi-Experimental Study | No information on protective factors of psychological resilience or risk factors for psychological resilience in mothers |
| Spallek et al. (2021) | Association of maternal migrant background with inflammation during pregnancy - Results of a birth cohort study in Germany | No information on protective factors of psychological resilience or risk factors for psychological resilience in mothers |
| Stanhope et al. (2021) | Perceptions of stress and resilience among Latina women enrolled in prenatal care in Metro Atlanta through an ecosocial lens | Not focused on forcibly displaced mothers or information on mothers cannot be distinguished from the study sample (e.g., parents, offspring, women) |
| Stewart et al. (2018) | Supporting refugee parents of young children: "knowing you're not alone" | Not focused on forcibly displaced mothers or information on mothers cannot be distinguished from the study sample (e.g., parents, offspring, women) |
| Tajima et al. (2010) | Parenting Beliefs and Physical Discipline Practices Among Southeast Asian Immigrants: Parenting in the Context of Cultural Adaptation to the United States | No information on protective factors of psychological resilience or risk factors for psychological resilience in mothers |
| Tariq et al. (2016) | "It pains me because as a woman you have to breastfeed your baby": decision-making about infant feeding among African women living with HIV in the UK | Not focused on forcibly displaced mothers or information on mothers cannot be distinguished from the study sample (e.g., parents, offspring, women) |
| Taylor et al. (2022) | Family support and mental health of Latinx children in migrant farmworker families | No information on protective factors of psychological resilience or risk factors for psychological resilience in mothers |
| Tingvold et al. (2012) | Seeking balance between the past and the present: Vietnamese refugee parenting practices and adolescent well-being | Not focused on forcibly displaced mothers or information on mothers cannot be distinguished from the study sample (e.g., parents, offspring, women) |
| Trachtenberg et al. (1995) | Portraits of success: Immigrant women's strategies for overcoming barriers in the urban United States | Not focused on forcibly displaced mothers or information on mothers cannot be distinguished from the study sample (e.g., parents, offspring, women) |
| Tulpule et al. (2022) | Differences in infant feeding practices between Indian-born mothers and Australian-born mothers living in Australia: a cross-sectional study | No information on protective factors of psychological resilience or risk factors for psychological resilience in mothers |
| Tunstall et al. (2010) | Residential mobility in the UK during pregnancy and infancy: Are pregnant women, new mothers and infants 'unhealthy migrants'? | No information on protective factors of psychological resilience or risk factors for psychological resilience in mothers |
| Valdez et al. (2013) | Feasibility, Acceptability, and Preliminary Outcomes of the Fortalezas Familiares Intervention for Latino Families Facing Maternal Depression | No information on protective factors of psychological resilience or risk factors for psychological resilience in mothers |
| Van Lieshout et al. (2011) | Assessing the measurement invariance of the Center for Epidemiologic Studies Depression Scale across immigrant and non-immigrant women in the postpartum period | No information on protective factors of psychological resilience or risk factors for psychological resilience in mothers |
| Vargas (2018) | Acculturation, parental involvement, and maternal self-efficacy: A qualitative study among Latin American women in the United States | Not focused on forcibly displaced mothers or information on mothers cannot be distinguished from the study sample (e.g., parents, offspring, women) |
| Velie et al. (2006) | Understanding the increased risk of neural tube defect-affected pregnancies among Mexico-born women in California: immigration and anthropometric factors | No information on protective factors of psychological resilience or risk factors for psychological resilience in mothers |
| Villegas et al. (2019) | The Effects of Hispanic Immigrant Mother's Resiliency on Children's Dietary Adjustment | No information on protective factors of psychological resilience or risk factors for psychological resilience in mothers |
| Villegas et al. (2019) | The effects of Hispanic immigrant mother’s resiliency on children’s dietary adjustment': Correction | duplicate |
| Walther et al. (2021) | A qualitative study on resilience in adult refugees in Germany | Not focused on forcibly displaced mothers or information on mothers cannot be distinguished from the study sample (e.g., parents, offspring, women) |
| Werneck et al. (2008) | Early Childhood Caries and Access to Dental Care among Children of Portuguese-Speaking Immigrants in the City of Toronto | Not focused on forcibly displaced mothers or information on mothers cannot be distinguished from the study sample (e.g., parents, offspring, women) |
| Williamson et al. (2014) | A Pilot Randomized Trial of Community-Based Parent Training for Immigrant Latina Mothers | No information on protective factors of psychological resilience or risk factors for psychological resilience in mothers |
| Xue et al. (2024) | Risk and Protective Processes in the Link Between Racial Discrimination and Chinese American Mothers' Psychologically Controlling Parenting | No information on protective factors of psychological resilience or risk factors for psychological resilience in mothers |
| Zúñiga and Belén (2020) | The power of our story: Strength of Latinx immigrant parents despite legal violence | No information on protective factors of psychological resilience or risk factors for psychological resilience in mothers |

## Table S2: Records Excluded at the Full-text Screening Stage, with Reasons – Updated Search.

| **Authors (Year)** | **Title** | **Exclusion reason** |
| --- | --- | --- |
| Baran et al. (2024) | Superhero in a skirt: Psychological resilience of Ukrainian refugee women in Poland. A thematic analysis | Not focused on forcibly displaced mothers or information on mothers cannot be distinguished from the study sample (e.g., parents, offspring, women) |
| Çakır (2025) | Factors and mechanisms of resilience among Turkish migrant women in the UK | Not focused on forcibly displaced mothers or information on mothers cannot be distinguished from the study sample (e.g., parents, offspring, women) |
| Dryjanska et al. (2025) | Resilience Against All Odds: How Refugee Women From Ukraine Find Courage Through Transnational Families | Not focused on forcibly displaced mothers or information on mothers cannot be distinguished from the study sample (e.g., parents, offspring, women) |
| Gargano et al. (2024) | What words can tell us about social determinants of mental health: A multi-method analysis of sentiment towards migration experiences and community life in Lima, Perú | Not focused on forcibly displaced mothers or information on mothers cannot be distinguished from the study sample (e.g., parents, offspring, women) |
| Golmohammad et al. (2025) | Undocumented Afghan refugee women's lived experiences of distress in Iran: A narrative inquiry of social suffering during the COVID-19 pandemic | Not focused on forcibly displaced mothers or information on mothers cannot be distinguished from the study sample (e.g., parents, offspring, women) |
| Gonzalez-Guarda et al. (2024) | Trajectories of physiological stress markers over time among Latinx immigrants in the United States: Influences of acculturative stressors and psychosocial resilience | Not focused on forcibly displaced mothers or information on mothers cannot be distinguished from the study sample (e.g., parents, offspring, women) |
| Guidi et al. (2025) | Hearing the voices of Ukrainian refugee women in Italy to enhance empowerment interventions | Not focused on forcibly displaced mothers or information on mothers cannot be distinguished from the study sample (e.g., parents, offspring, women) |
| Hu et al. (2024) | A Randomized Controlled Trial of Two Parent-Child Parallel Interventions to Enhance Positive Adaptation of Immigrant Families in Hong Kong: The Moderating Role of Depressive Symptoms | Not focused on forcibly displaced mothers or information on mothers cannot be distinguished from the study sample (e.g., parents, offspring, women) |
| Jiménez-Lasserrotte et al. (2024) | Understanding resilience among migrant women in a humanitarian reception center: a qualitative study | Not focused on forcibly displaced mothers or information on mothers cannot be distinguished from the study sample (e.g., parents, offspring, women) |
| Khailenko and Bacon (2024) | Resilience, avoidant coping and post-traumatic stress symptoms among female Ukrainian refugees and internally displaced people | Not focused on forcibly displaced mothers or information on mothers cannot be distinguished from the study sample (e.g., parents, offspring, women) |
| Lindert et al. (2023) | Factors Contributing to Resilience Among First Generation Migrants, Refugees and Asylum Seekers: A Systematic Review | Review, commentary papers and research protocols (i.e., not reporting novel data/findings) |
| Liu (2024) | Cultural models of shyness and parenting among local and immigrant mothers in Hong Kong | No information on protective factors of psychological resilience or risk factors for psychological resilience in mothers |
| Nguyen-Nalpas (2025) | Tracing reverberations: Vietnamese refugee mothers' adaptations to complex structural forces and intergenerational engagements | No information on protective factors of psychological resilience or risk factors for psychological resilience in mothers |
| Schuster (2024) | Gendered effects of climate change and health inequities among forcibly displaced populations: Displaced Rohingya women foster resilience through technology | Review, commentary papers and research protocols (i.e., not reporting novel data/findings) |
| Seff et al. (2024) | Women's collectives and social support: exploring pathways and impacts among forcibly displaced women | Not focused on forcibly displaced mothers or information on mothers cannot be distinguished from the study sample (e.g., parents, offspring, women) |
| Shafiq et al. (2025) | Toward Resilient Maternal, Neonatal and Child Health Care: A Qualitative Study Involving Afghan Refugee Women in Pakistan | Not focused on forcibly displaced mothers or information on mothers cannot be distinguished from the study sample (e.g., parents, offspring, women) |
| Son et al. (2025) | Factors Influencing eHealth Literacy Related to Parenting Among Asian Immigrant Mothers in South Korea | No information on protective factors of psychological resilience or risk factors for psychological resilience in mothers |
| Sousa et al. (2023) | "Life becomes about survival": Resettlement, integration, and social services among refugee parents | Not focused on forcibly displaced mothers or information on mothers cannot be distinguished from the study sample (e.g., parents, offspring, women) |
| Sun et al. (2024) | All in the Family: The Complementary Protective Roles of Spousal and Other Family Support for Chinese Immigrant Mothers' Life Satisfaction Over Time | No information on protective factors of psychological resilience or risk factors for psychological resilience in mothers |
| Tse et al. (2025) | Parallel Ethnic Identity Development of Mexican-Origin Adolescents and Mothers Under the Influence of Neighborhood Latinx Concentration and Ethnic-Racial Diversity | No information on protective factors of psychological resilience or risk factors for psychological resilience in mothers |
| Vasquez (2025) | Unveiling sources of resilience and social support dimensions: Honoring Mexican immigrant experiences and their migration journeys in a borderlands community based participatory project | Not focused on forcibly displaced mothers or information on mothers cannot be distinguished from the study sample (e.g., parents, offspring, women) |
| Verschuuren et al. (2024) | Suboptimal factors in maternal and newborn care for refugees: Lessons learned from perinatal audits in the Netherlands | No information on protective factors of psychological resilience or risk factors for psychological resilience in mothers |
| Walsh et al. (2024) | Risk, resilience and family relationships among at-risk Ethiopian immigrant youth in Israel: A focus group investigation | No information on protective factors of psychological resilience or risk factors for psychological resilience in mothers |
| Yu et al. (2024) | Applying Kumpfer's resilience framework to understand the social adaptation process of the trailing parents in China | Not focused on forcibly displaced mothers or information on mothers cannot be distinguished from the study sample (e.g., parents, offspring, women) |
